# Human resources and models of mental healthcare integration into primary and community care in India: Case studies of 72 programmes

**DOI:** 10.1371/journal.pone.0178954

**Published:** 2017-06-05

**Authors:** Nadja van Ginneken, Meera S. Maheedhariah, Sarah Ghani, Jayashree Ramakrishna, Anusha Raja, Vikram Patel

**Affiliations:** 1 Centre for Global Mental Health, London School of Hygiene and Tropical Medicine, London, United Kingdom; 2 Sangath, Porvorim, Goa, India; 3 Department of Health Services Research, Institute of Psychology, Health and Society, University of Liverpool, Liverpool, United Kingdom; 4 National Institute of Mental Health and NeuroSciences, Bengaluru, Karnataka, India; 5 Yale University, New Haven, Connecticut, United States of America; 6 Harvard Medical School, Boston, Massachusetts, United States of America; Yokohama City University, JAPAN

## Abstract

**Background:**

Given the scarcity of specialist mental healthcare in India, diverse community mental healthcare models have evolved. This study explores and compares Indian models of mental healthcare delivered by primary-level workers (PHW), and health workers’ roles within these. We aim to describe current service delivery to identify feasible and acceptable models with potential for scaling up.

**Methods:**

Seventy two programmes (governmental and non-governmental) across 12 states were visited. 246 PHWs, coordinators, leaders, specialists and other staff were interviewed to understand the programme structure, the model of mental health delivery and health workers’ roles. Data were analysed using framework analysis.

**Results:**

Programmes were categorised using an existing framework of collaborative and non-collaborative models of primary mental healthcare. A new model was identified: the specialist community model, whereby PHWs are trained within specialist programmes to provide community support and treatment for those with severe mental disorders. Most collaborative and specialist community models used lay health workers rather than doctors. Both these models used care managers. PHWs and care managers received support often through multiple specialist and non-specialist organisations from voluntary and government sectors. Many projects still use a simple yet ineffective model of training without supervision (training and identification/referral models).

**Discussion and conclusion:**

Indian models differ significantly to those in high-income countries—there are less professional PHWs used across all models. There is also intensive specialist involvement particularly in the community outreach and collaborative care models. Excessive reliance on specialists inhibits their scalability, though they may be useful in targeted interventions for severe mental disorders. We propose a revised framework of models based on our findings. The current priorities are to evaluate the comparative effectiveness, cost-effectiveness and scalability of these models in resource-limited settings both in India and in other low- and middle- income countries.

## Introduction

In low- and middle- income countries (LMICs) very few mentally ill people receive formal mental healthcare. This treatment gap persists due to scarce specialist resources and large inequities and inefficiencies in resource allocation [1, 2]. To achieve universal health coverage, task-sharing and better leadership have been advocated [3]. Within mental healthcare, the World Health Organisation’s (WHO) Mental Health Gap Action Programme published guidelines for mental health interventions delivered by primary care (PC) doctors and nurses [4]. There is now growing evidence for cost-effective and feasible care [5] and for the effectiveness of primary-level health workers (PHWs) in providing mental health interventions [6]. PHWs include professionals (primary-level doctors, non-physician clinicians and social workers), and lay health workers (LHWs), who are not mental health specialists but have received minimal training in mental healthcare.

A comprehensive Indian mental health workforce has not yet been achieved. There is currently a 40- to 60-fold deficit in psychiatrists, and even fewer psychologists, psychiatric social workers and psychiatric nurses [7]. In addition, only about a sixth of 640 districts have implemented the District Mental Health Programme (DMHP), which is India’s national effort to decentralise mental healthcare to promote early detection and stigma reduction and to improve accessibility of care (notably through primary care). In addition, within these implementing districts, many PC doctors remain untrained [8]. Since the 1990s and in response to local needs, non-governmental organisations (NGOs) have emerged with innovative models of PHW-delivered mental healthcare [9, 10].

This study’s aim is to describe and compare current Indian models of PHW-delivered mental healthcare in India, which to date has not been done comprehensively. Exploring these models and their human resources is important to identify innovative strategies that could be implemented at scale. A further paper on the same data set will explore health workers’ perceptions of their work’s acceptability and sustainability.

**Fig 1:**
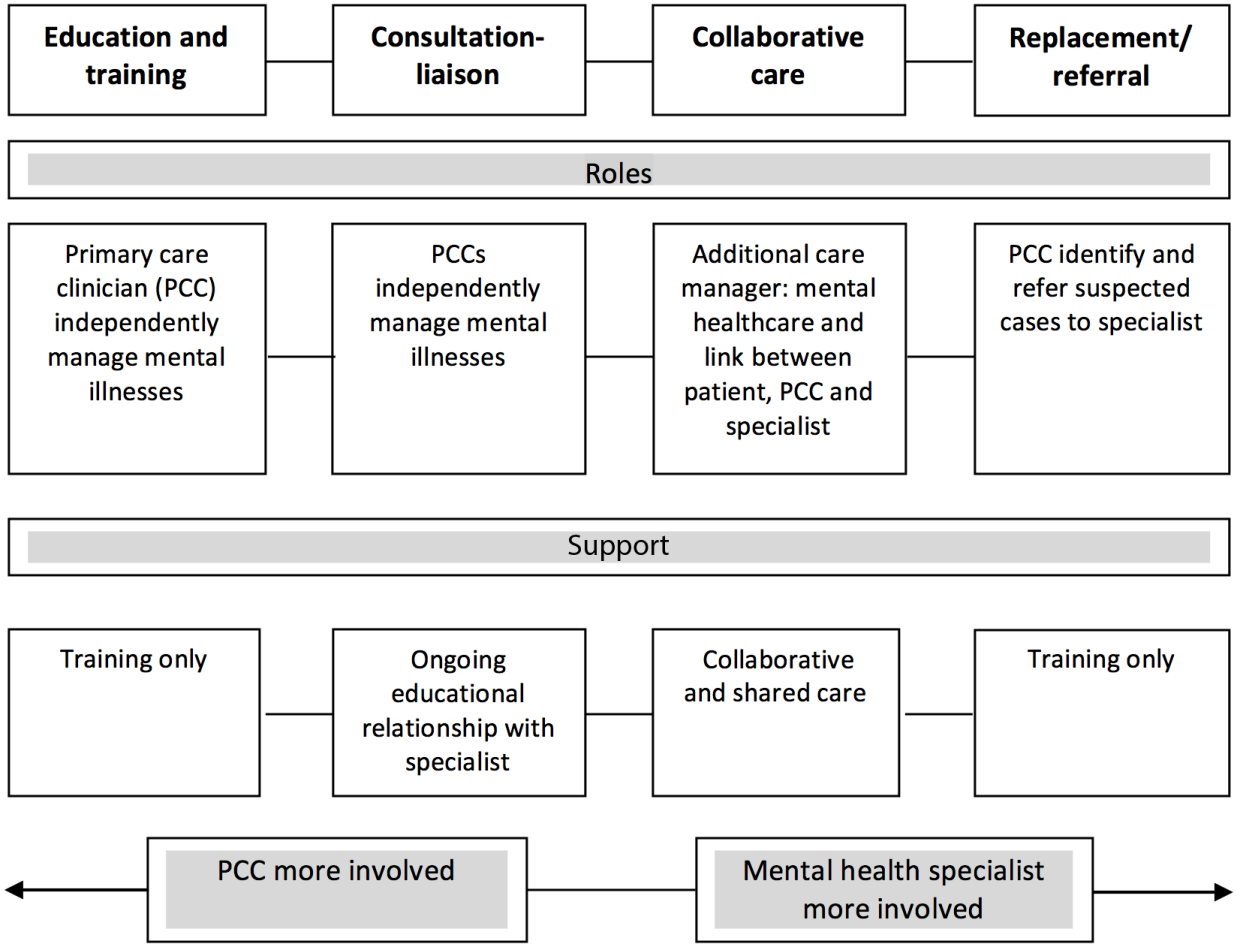
Models of primary mental healthcare (adapted from [12]).

Several established frameworks exist to categorise integrative primary mental healthcare [11–15]. The Bower framework was chosen as it best applied to LMICs context (Fig 1). It describes models according to the level of specialist involvement needed within primary care from little (training) to much (identification and referral). This is a main concern in LMICs where specialist resources are scarce. It also encompasses collaborative care, which is currently viewed as the gold standard model. Collaborative care is defined as complex interventions usually involving the addition of a care manager, (a new ‘linking’ cadre between PC provider, specialist and patient, with clinical responsibilities [16]). It has convincing evidence of effectiveness for many chronic disorders such as depression [6, 17, 18], anxiety [17] and combined diabetes and depression [19].

## Methods

### Study setting

We identified 122 potential organisations (organised groups working on first-level health/mental healthcare provision) through snowballing and web searches. DEVnet and Google.in were searched for the following terms: primary, community, mental, depression, psychosis, neurological, epilepsy substance abuse, alcohol, drugs. In light of a mixed private and government healthcare system in India, the Alma Ata definition of primary healthcare was used: “the first level of contact of individuals, the family and community” involving the health sector and related sectors [20].

**Fig 2:**
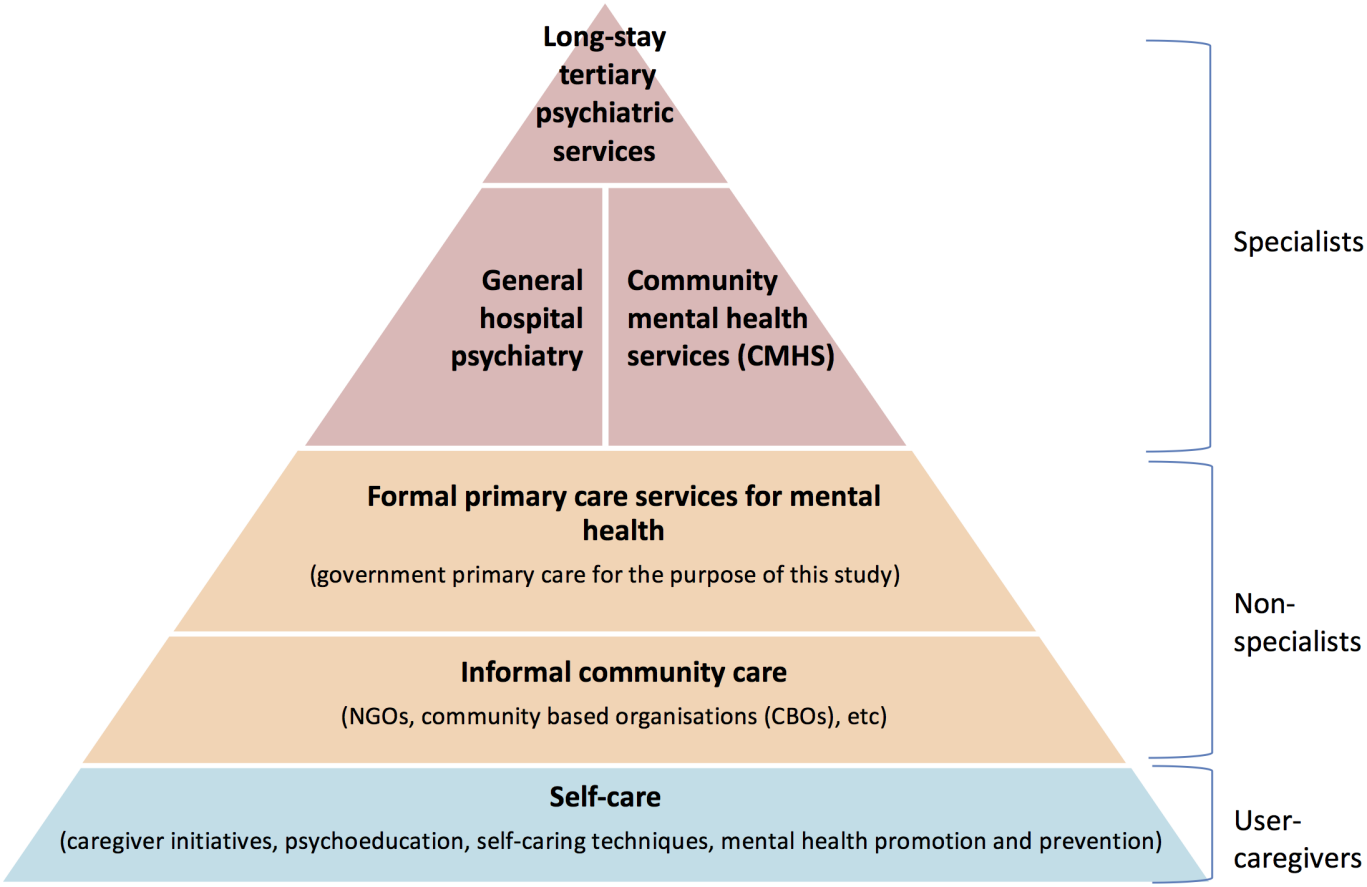
Organisation of mental health services (adapted from WHO-WONCA 2008).

**Fig 3:**
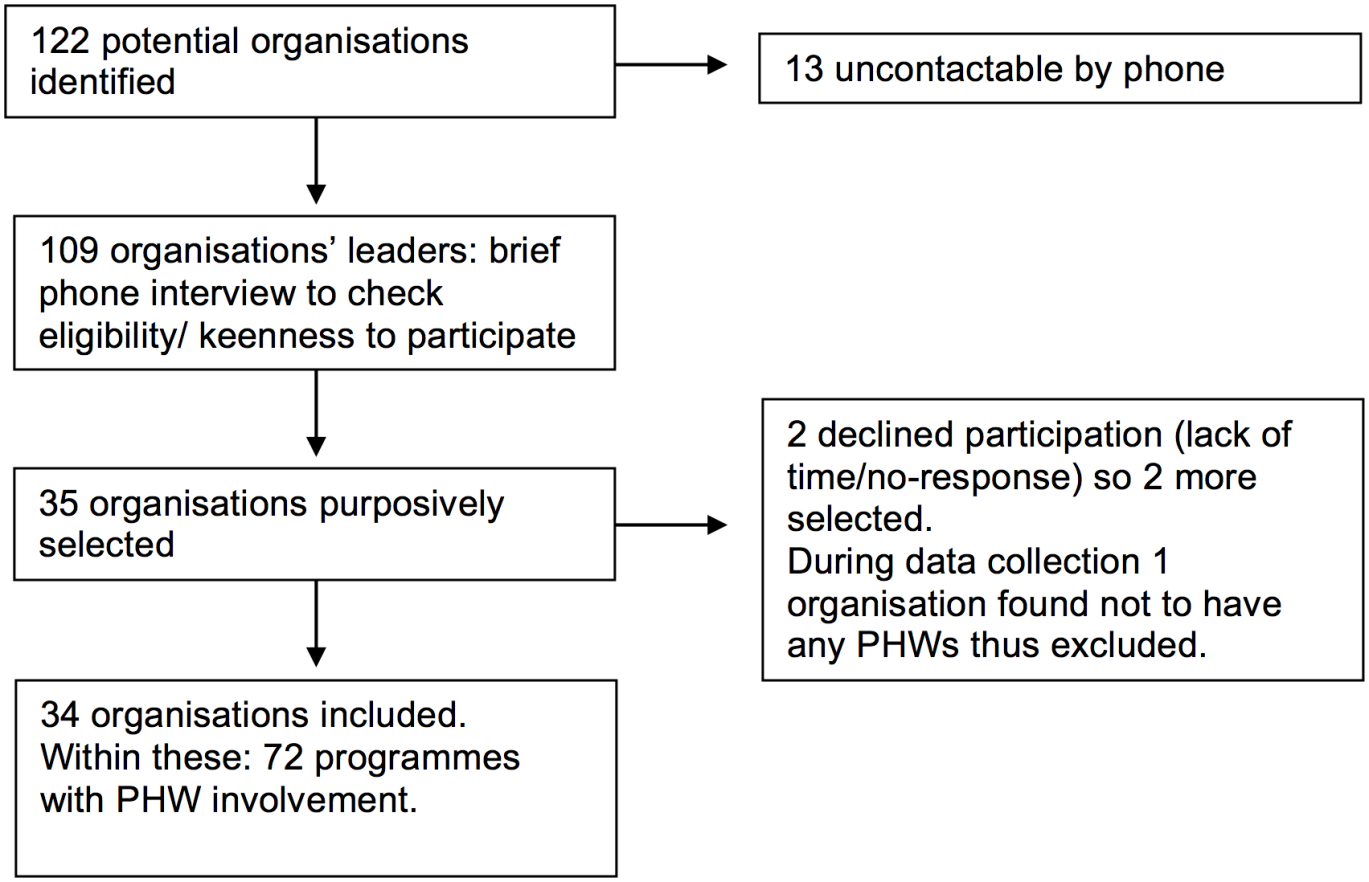
Flow diagram of case study selection.

Our inclusion criteria thus included government primary care and private not-for-profit (e.g. NGOs) primary- and community care (Fig 2) [15]. These organisations had to address one or more mental, neurological and substance-use (MNS) disorders, have at least one PHW cadre and have been working for a minimum of two years. NGOs were selected purposively to represent different population characteristics, PHWs types and roles, and service delivery models. DMHP sites were eligible only if they had an active primary care programme. Their further selection was opportunistic according to agreeability (no response from most) and proximity (the research team in Karnataka could meet relevant authorities to establish contractual agreements). We excluded private-for-profit organisations as their business model to maximise profits may become more important than healthcare provision [3]. Fig 3 illustrates how the case studies were selected. Organisations had between one and eight programmes (sets of activities) which utilised different delivery models, hence the selection of 34 organisations resulted in 72 programmes. Each programme became a case study.

### Sampling

Across the 72 programmes, 246 health staff were interviewed (Table 1). Programme leaders/coordinators chose representative staff for interviews. In all programmes we interviewed at least one PHW and either a coordinator, leader and/or specialist. Leaders/specialists sometimes worked across programmes within an organisation hence their interviews included information on several programmes.

**Table 1:** Interview characteristics.

Data collection	Details				Total
**Participants**	PHWs (134); (mental) health coordinators (33); specialists (40); leaders (34), other programme staff (e.g. pharmacist, admin) (5)				246
**Interviews**		Recorded	Non-recorded	Subtotal	125
	Semi-structured interviews	66	30	96	
	Focus groups	24	5	29	
	Subtotal	90	35	125	
**Translations**	Mizo (1); Oriya (1); Malayalam (2); Telugu (4); Tamil (4); Hindi (9); Kannada (23)				44

### Data collection

A case study approach was adopted to describe a real world setting and to understand successful or challenging factors [21]. Data were collected between 2010 and 2012. Case studies involved semi-structured interviews (with coordinators, managers and specialists), focus groups (with PHWs, and on occasion with their supervisors) and visits to all programmes, to achieve depth of information. These data were collected by NvG, MM or SG in English, Kannada and Hindi (Table 1). NvG (family physician and PhD candidate) and MM (research coordinator with a PhD in anthropology) were experienced qualitative researchers. SG (research assistant with a Masters in psychology) received qualitative training and supervision from NvG and MM. Interpreters (allied project staff) were sourced locally for other languages. Interviews/focus groups were recorded and transcribed and translated by professional translators with prior experience in community health research projects. All the Kannada and Hindi transcriptions and translations were checked for accuracy by bilingual researchers (MM and SG respectively). Noisy environments or participant refusal prohibited some recordings—copious notes in English were taken instead. Questions were adapted from an existing case-study methodology for community mental health programme evaluations in low-income countries [22]. Health workers (non-specialists and specialists) were asked to describe their activities, roles and barriers/solutions. Founders or programme managers were asked about programme characteristics (e.g. funding, management), views on PHWs and future plans and project scalability (S1 Text).

Observations of the location and infrastructure during site visits were recorded in summary sheets (S1 and S2 Text). Published and unpublished documentary sources were sought from participants to complement and corroborate interview data. Little quantitative data existed (apart from occasional annual reports, leaflets and few published material) therefore most information was drawn from interview data. These complementary methods allowed triangulation to achieve maximal programme information. No inconsistencies between data were observed.

Ethical approval was obtained from the London School of Hygiene and Tropical Medicine, Sangath and the Indian Council of Medical Research. Participants provided written consent. Consent forms and information sheets were translated into Hindi and Kannada by independent researchers and back-translated by MM and SG.

### Data analysis

Framework analysis was chosen as it focusses on generating policy- and practice-oriented findings. It retains the integrity of interviewees’ narratives whilst also classifying and comparing themes [23]. A thematic coding framework was created in NVIVO for transcripts, notes and summary sheets to structure multiple-researcher coding (NvG, MM, SG and JR) [24]. We corroborated documentary sources with these latterly. We piloted and revisited the framework with JR. VP and JR (both Indian professors with qualitative expertise) provided methodological support and contextual interpretation during analysis. The lead researcher (NvG) cross-checked 15 interviews for consistency and reliability across researchers by recoding and qualitatively comparing codes [24]. Ninety-two percent of codes overlapped (given researchers were coding from the same framework as described above) which suggests good agreement was achieved.

The factual programmatic data gathered was taken at face value to represent the ‘truth’. This post-positivist approach is possible within content analysis [23]. It was justified as there was sparse documentary evidence [25]. Cross-checking different workforce members’ reports for inconsistencies and completeness, and getting organisations’ validation of summary factual reports submitted to them ensured maximal factual accuracy [23]. Deviant case analysis and data triangulation increased internal data validity and credibility of conclusions.

The coding framework was charted into tables to compare different features (health worker and programme characteristics) across programmes. Patterns were mapped between different human resources and programme features [23].

## Results

**Fig 4:**
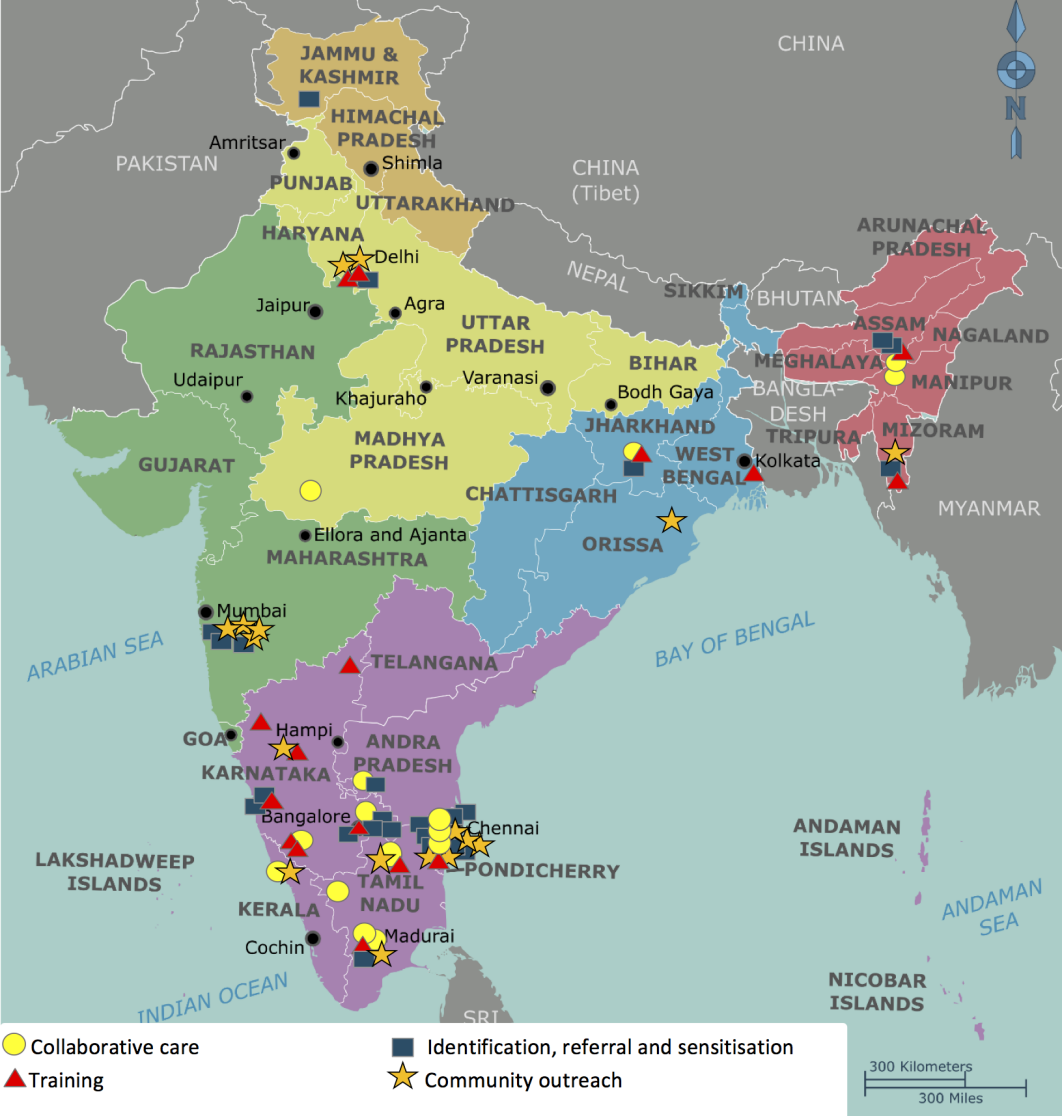
Location of the 72 programmes. (adaption of map by Cacahuate, Ravikiran Rao, Nichalp, CC BY-SA 4.0, via Wikimedia Commons).

The 72 PHW programmes were selected across 12 states (Fig 4, S1–S4 Tables). We categorised programmes according to whether or not they were collaborative care models and where they fitted within the Bower framework (Fig 1). The non-collaborative programmes fitted into four categories. Three categories are adapted from the Bower framework: 1) training, 2) consultation liaison and 3) identification, referral and sensitisation models. Several programmes did not fit these categories therefore a new category was created: the community outreach model. Below we describe and analyse the features of programmes’ models, and the types and roles of their human resources.

### The collaborative care model

#### A model with many variations

**Fig 5:**
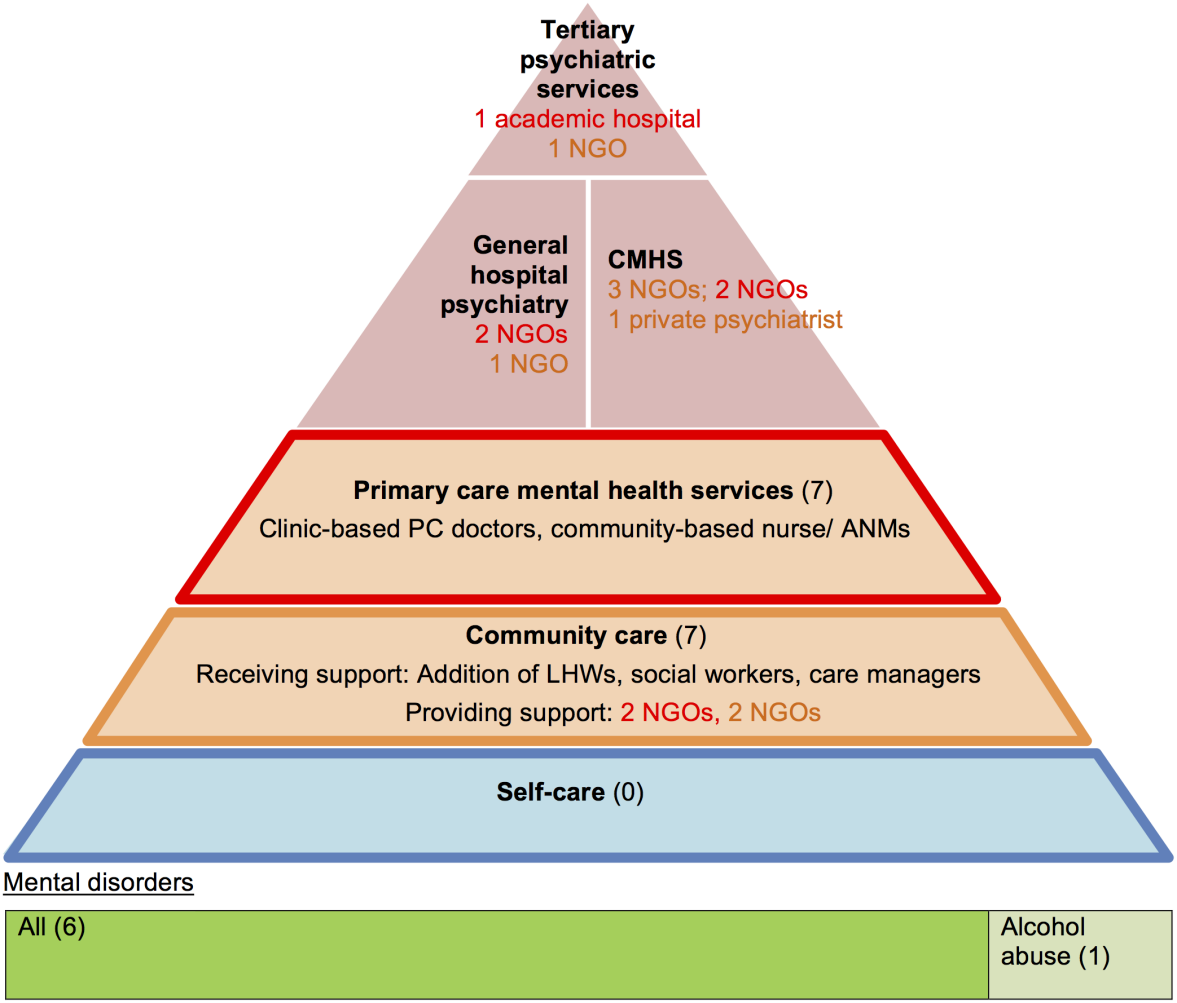
Collaborative care models which utilise primary care and community care: characteristics and relationships (n = 7). The colour of the font represents which non-specialist level (primary-, community- or self-care) the organisation supports. CMHS: community mental health services; ANM: Auxiliary Nurse Midwife (a salaried government PC LHW); NGO: non-governmental organisation.

**Fig 6:**
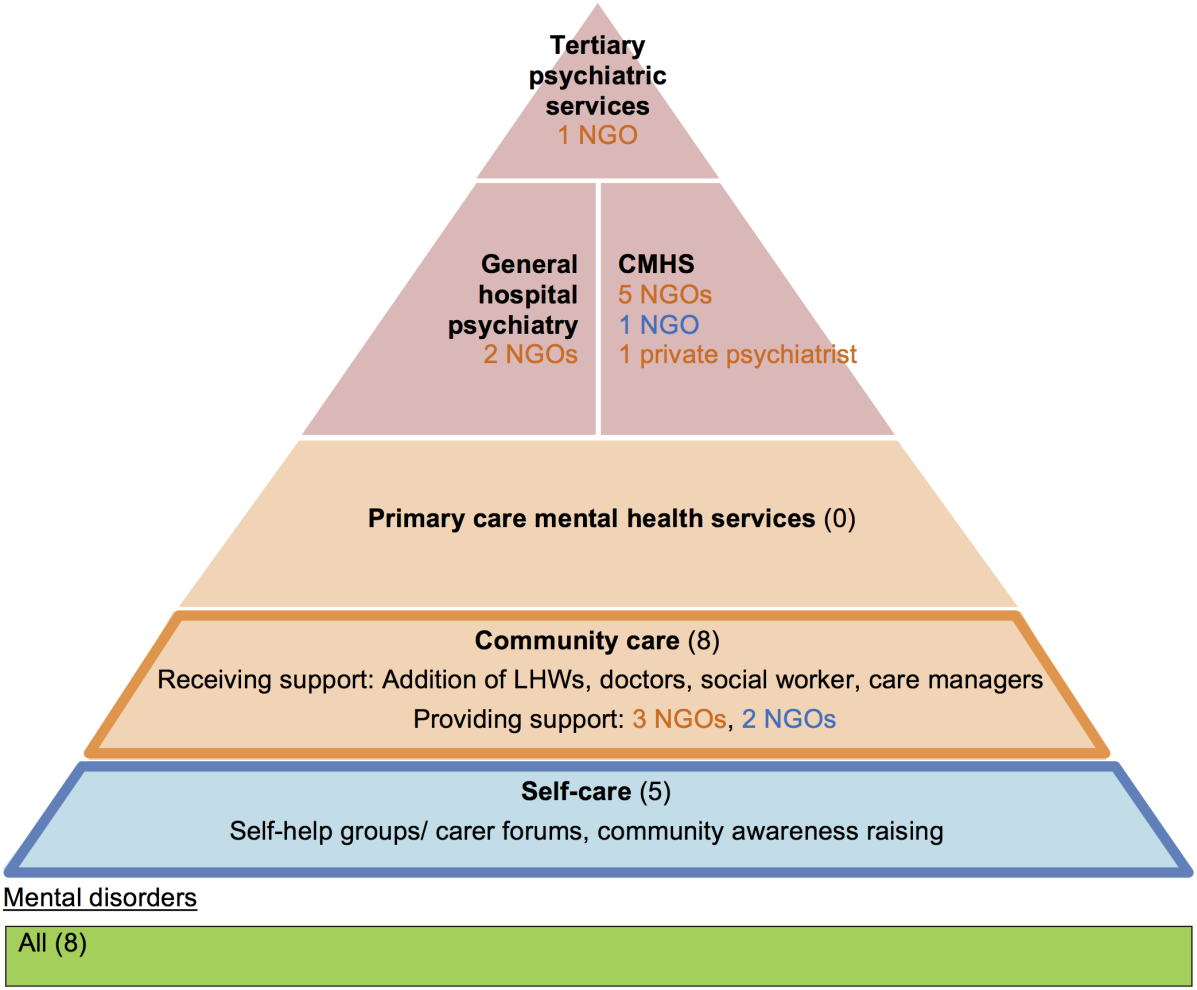
Collaborative care models which utilise community care only: characteristics and relationships (n = 8). The colour of the font represents which non-specialist level (primary-, community- or self-care) the organisation supports. CMHS: community mental health services; LHW: Lay health worker.

Collaborative care was implemented by 15 NGOs. These complex interventions, most of which covered all mental disorders, involved NGO-led multi-disciplinary teams (MDTs) and shared care between different organisations. Half of them also involved government primary care. Figs 5 and 6 show that support and supervision did not come solely from specialists. Experienced PHWs in mental healthcare within community organisations also offered support to other non-specialist sectors (primary- and self-care). These collaborations also involved multiple NGO partnerships (Box 1 example 1).

**Box 1. Collaborative care examples.**

**Example 1: Collaborative care involving multiple collaborations and coordinators. BasicNeeds-UK (Jharkhand)**

The international mental health NGO BasicNeeds-UK has partnered with a developmental NGO, NBJK (which coordinates activities with several community-based organisations (CBOs)). The BasicNeeds-UK mental health coordinator provides technical support to NBJK for the mental health programme. Within NBJK the programme manager liaises with the care manager who links specialist-LHW care, and trains/supervises LHWs. LHWs include NBJK LHW volunteers (who identify, refer, follow-up, raise awareness and provide psychosocial support), clinic administrative volunteers and CBO volunteers (initiate livelihood activities, care and psychoeducation). Psychiatrists from RINPAS (a government psychiatric hospital) perform monthly outreach clinics (clinical input only).

**Example 2: Collaborative care in PC settings. The Banyan rural mental health programme (Tamil Nadu)**

The Banyan NGO set up a PC centre staffed by a PC doctor. This doctor’s consultations focus on general medical issues; he has not engaged with mental healthcare yet (despite Banyan’s encouragement). They also have a community outreach team with LHWs (awareness, detection, follow-up, psychosocial support, counselling). LHWs have intensive daily supervision by a care manager (who also does patient-specialist-LHW liaison) and do joint visits with social workers and the care manager. A Banyan-employed psychiatrist and psychologist conduct outreach clinics (diagnose, treat and supervise care-manager and LHWs regularly). This facility also offers vocational training for rehabilitating patients with severe mental illnesses.

#### Complexity of human resources

Care managers had complex and varied coordination and clinical roles. In some programmes, the care manager and clinician were the one person (3 programmes). In others, several layers of coordinators were identified (e.g. non-clinical organisational coordinators as well as clinical coordinators). Care managers were mainly experienced PHWs and were supervised regularly (Table 2, S1 Table).

**Table 2:** Collaborative care human resources.

Health workers and backgrounds	Nb of pro-grammes	Roles	Training and supervision
**Doctors**: Primary care (PC) doctors unless otherwise specified	12	exclude organic disease/ general healthcare (6) identification, referral, follow-up (4) psychiatric diagnosis/ treatment by community gynaecologists (2)/ PC doctor (2) counselling (1)	ad hoc supervision and ongoing training (10) weekly supervision (1- gynaecologist)
**Non-physician professionals**: 4 social workers (SW); 1 nurse	4	outreach work (identify, refer, follow-up, facilitate rehab activities) supervise LHWs/ care managers counselling (Banyan RMHP)	regular by psychiatrist (4), psychologist (4) or primary health-worker (PHW) coordinator (1)
**Lay health workers (LHWs)**: primary/ secondary school (13); graduate (1-GASS)	14	identification, referral sensitisation medication adherence psychosocial support counselling (7) facilitate income generating/self-help groups/patient advocacy (4) conducting surveys (1) bring patients to camps (4)	Training: by specialists Supervision: by care managers
**Community members**: community leaders/members (MICP, NBJK, Ashwini); self-help groups/forums (Ashagram, SACRED)	5	identification/ referral (4) general support, patient advocacy (5)	ad hoc by coordinators
**Care managers (CMs)**: experienced LHWs (8); graduates/SW (4); gynaecologist (2); psychiatrist (1)	15	patient-LHW-specialist liaison coordinate activities clinical roles (not all, some had none); train and supervise LHWs	Training: by specialists Supervision: LHW CMs: by SW, psychiatric social worker (PSW) or graduate coordinator professional CMs: by psychiatrist /head
**Training and administrative coordinators**: graduates	5	coordinate programme activities (5) coordinate training (1) train/supervise PHWs/community members (3)	by specialists
**Specialists** (team structure): specialist MDTs i.e. psychiatrist +/-psychologist+/-PSW (7); linked psychiatrist (6); no psychiatrist (for referral only) (1) (have psychologist/PSW)	15	outreach clinics (12) counselling (by PSW) (1) PHW training (9) supervision of professional PHW (5) or care manager (5) organisation leadership (3 psychiatrists, 3 psychologist/PSWs)	none

Banyan RMHP: rural mental health programme; GASS: Grameena Abhyudaya Seva Samasthe—community based rehabilitation (CBR) workers programme; MICP: Mallapuram initiative in Community Psychiatry; NBJK: Nav Bharat Jagrath Kendra.

The most predominant PHWs used were LHWs, supervised by specialists. They featured in every programme except for one (the Banyan-Family Planning Association mental health programme used a community gynaecologist). LHWs performed psychosocial interventions to complement physical health which was addressed at PC level (Box 1 example 2). Their detailed roles are listed in Table 2. Interestingly, NGO-employed PHWs in non-PC settings encouraged self-care and development (Fig 6) whereas PC PHWs did not (Fig 5). Broader expectations of NGO PHWs are reflected in their longer training (3–12 days with refresher training) and adequate incentivisation compared to government LHWs (1–2 days training and no incentivisation for their mental health roles).

Generalist doctors were seldom utilised for mental health roles. Four programmes expected them to diagnose and treat mental illness, though with Ashwini and ‘the ANT’ this could be explained by the co-founders being respectively a gynaecologist and a generalist doctor. Otherwise PC doctors excluded organic disorders or identified and referred to a specialist (Table 2). The dearth of PC doctor usage was a result of a/ NGOs meeting resistance in engaging PC sites (such as Banyan: Box 1 example 2) and b/ programmes having psychiatric outreach clinics.

Specialist input remained significant in most programmes as psychiatrists usually first diagnosed patients, and then matched care according to their needs. They also provided initial training to most PHWs and supervised care managers. However where mental health initiatives had been appended to non-medical organisations (such as Ashadeep and Ashagram), external private psychiatrists were hired to deliver outreach clinics and had no input into supervision and training. They had a higher turnover. Specialist support intensity changed in many projects after 3–5 years. Three programmes had phased out specialist support once PHW follow-up mechanisms were established (e.g. Ashwini).

The next four models describe the features of non-collaborative care models used within other programmes.

### The training model

#### A simple and widespread model

**Fig 7:**
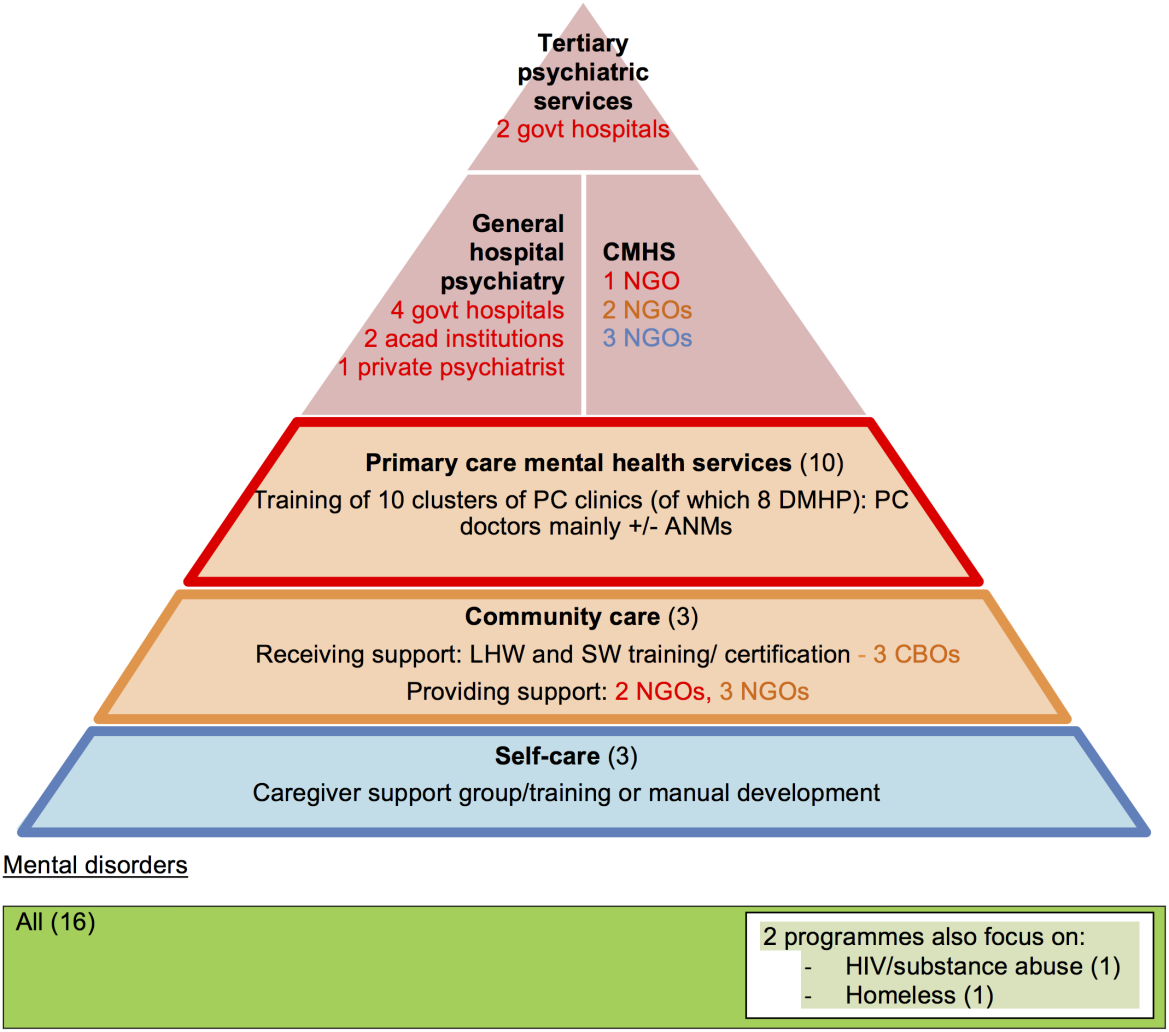
Training models—Characteristics and relationships (n = 16). The colour of the font represents which non-specialist level (primary-, community- or self-care) the organisation supports. CMHS: community mental health services; ANM: Auxiliary-Nurse Midwife; SW: social worker; LHW: lay health worker; PC: primary care; NGO: non-governmental organisation); CBO (community-based organisation); DMHP: District Mental Health Programme of India.

The numerous one-time training programmes could be categorised into: 1/ demand-driven NGO-to-NGO health worker training to broaden current care (clinical and networking skills) and self-care (e.g. caregiver training and manuals); and 2/ psychiatrist-driven training (from NGOs, government or individual private psychiatrists) to government PC doctors (Fig 7). This includes the government’s nationwide DMHP programme. As with collaborative care, the training came not just from specialist sectors but sometimes also from within non-specialist community care NGOs (Fig 7). Furthermore the retention of PC doctors threatened their sustainability. Most NGO-led PC doctor training programmes had closed as they found it futile due to the frequent transfer of doctors to other posts (S2 Table).

#### Insufficiently supported human resources

PC doctors generally had inadequate support for their additional mental health roles. Barriers identified included:

- PC doctors were not engaged in programme decision making
- They had no patient information sharing system with specialists
- They also had insufficient training: doctors were supposed to receive 15–30 days over 5 years but in reality only received 3 to 9 days
- There was inadequate supervision due to reduced psychiatrist availability (many DMHP psychiatrist posts were vacant), or the absence of a mental health coordinator (all apart from the DMHP-Karuna Trust public private partnership)
- Government drug supply was generally poor as was clinical information sharing.

NGOs continued providing one-off training in psychosocial support to LHWs (Table 3) but no mechanism was in place to evaluate the implementation (PHW competency and adherence to teachings) or impact of this training (S2 Table).

**Table 3:** Training model human resources.

Health workers and backgrounds	Nb of programmes	Roles	Training	Supervision
**PC doctors**	11	diagnose and treat refer educate patients	variable length: 9–30 days (Karnataka DMHP) 15 days (other DMHP) 3 days (closed NGO programmes)	programme implementation (not clinical) support (1)
**Non-physician professionals**: pharmacists (2 DMHP); community-based rehabilitation worker (CBR) (Samuha)	3	pharmacists: dispense drugs awareness-raising (2) CBR worker: social worker (SW) roles non-specific counselling	by specialists: ad hoc (DMHP) regular (Samuha)	by specialists: ad hoc (DMHP) regular (Samuha)
**LHWs:** literate (3); secondary school (3); graduate (1)	7	identification and referral only (DMHP LHWs) community sensitisation (NGOs) psychosocial interventions non-specific counselling (2)	by specialists	none
**Community members**: community leaders (1); anganwadis/ self-help groups (1); caregivers (3)	5	psychosocial support, self care referral networking/advocacy (community leaders) medical adherence (caregivers)	by specialists	none
**Training and administrative coordinators**: specialists (10); general doctor (1); (post)graduate (5)	16	training coordination	by specialists	none
**Specialists** (team structure): specialist multidisciplinary teams (MDT) (5); psychiatrist only (9); no psychiatrist (3) (have psychologist/ PSW in 2 of these)	all	clinical/outreach (MDT/psychiatrists) PC doctor/SW training (psychiatrists) LHW/caregiver training (by psychol/PSW) caregiver training (1). lead organisation (6)	none	none

DMHP: District Mental Health Programme

### The consultation-liaison model

No programme currently used the consultation-liaison model described in high income country (HIC) settings which provides ongoing educational support and helping with difficult cases [26]. However it was used as a stepping stone towards independent PC practice. For example the Karuna Trust had started psychiatrist and PC doctor co-consulting. Subsequently within their partnership with the government DMHP they practiced consultation-liaison for a year followed by withdrawing psychiatric help and adopting a DMHP training model.

### The identification, referral and sensitisation model

#### A widely used model

**Fig 8:**
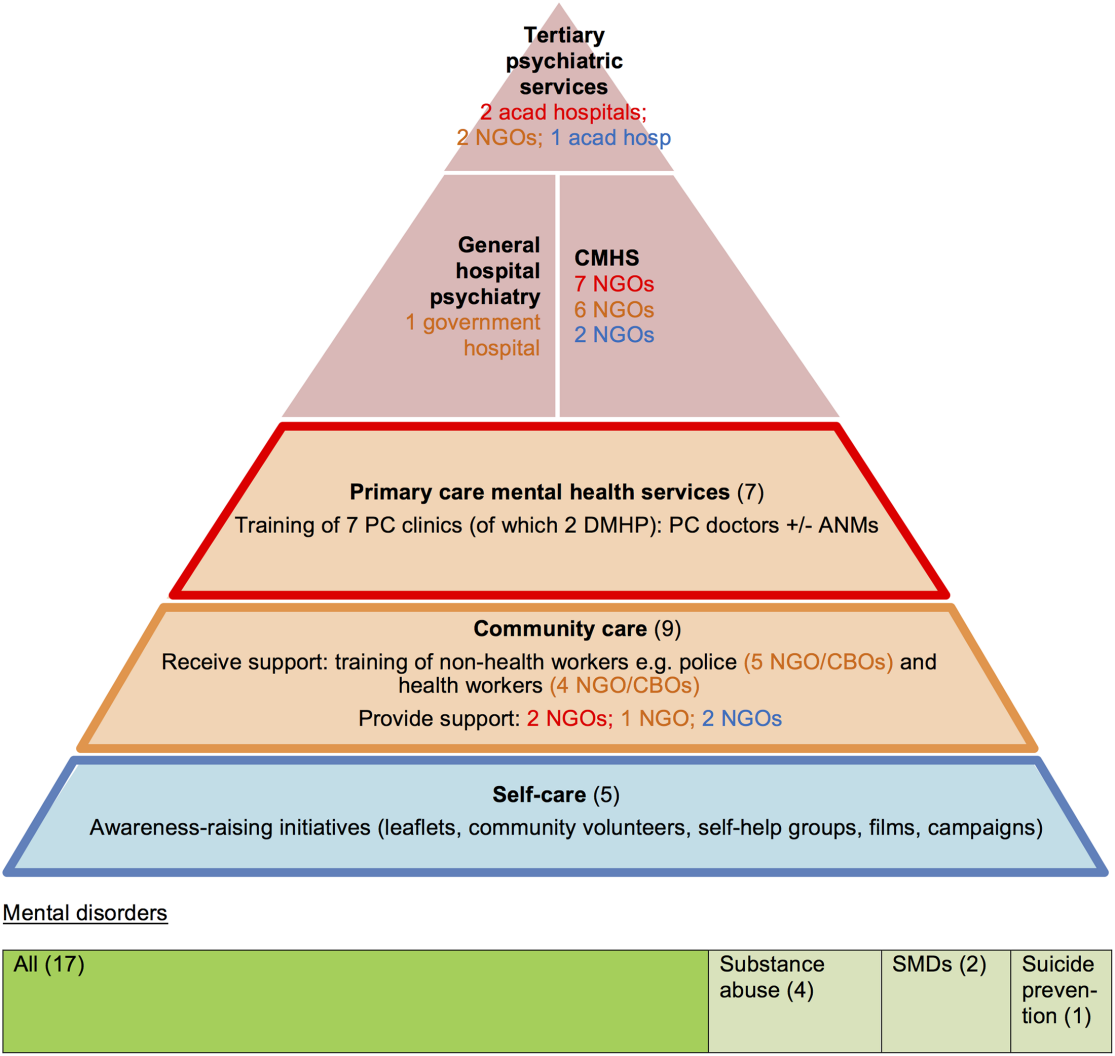
The identification and referral model: Characteristics and relationships (n = 24). The colour of the font represents which non-specialist level (primary-, community- or self-care) the organisation supports. CMHS: community mental health services; ANM: Auxiliary-Nurse Midwife; PC: primary care; NGO: non-governmental organisation; CBO: community-based organisation (smaller local organisations which do not have NGO status); DMHP: District Mental Health Programme of India; SMD: severe mental disorders.

A third of programmes used this model of training PHWs to identify and refer to specialists. We expanded the Bower replacement and referral model to include programmes which trained PHWs in broader processes of identification such as raising awareness on mental illness, providing referral pathways information and promoting self-care (Fig 8). We have thus renamed this model to include this breadth of coverage. As per above models, Fig 8 illustrates how training was received both from specialists and non-specialist community organisations.

Most programmes resembled the training programmes as they provided sporadic training to third party organisations, with usually no ensuing supervision or support (Table 4). Most of these had closed because of contracts not being renewed or because NGOs felt the training was ineffective (S3 Table). However training of own or partner PHWs was more sustainable as these training (and sometimes supervisory) activities were combined with other outreach activities, though no system existed to assess these programmes’ impact.

**Table 4:** Identification and referral human resources.

Health workers and backgrounds	Nb of pro-grammes	Roles	Training	Supervision
**PC doctors**	6	identification referral follow-up (2 current; 4 closed)	1–3 days (except 2 programmes: 7–15 days)	none
**Non-physician professionals:** students (social worker (SW) and nursing); nurse; pharmacist	2	Students (TTK): identify/ refer people with alcohol problems. Nurse, pharmacist (AIIMS): first aid assist doctor medication dispensing /counselling	one-off training	by PC doctor (AIIMS)
**LHWs**: literate (3); secondary school (7)	10	identification, referral, follow-up (all) sensitisation (3) psychosocial support (2)	1 day except GASS (5 days) and RFS (3 days)	none except NBJK and AIIMS (ad hoc)
**Community members**: community leaders (1); religious leaders (1); police/ other community workers (7); anganwadis/ self-help groups (2)	11	identification/ referral psychosocial support (2) campaigns/ sensitisation (9)	by specialists	none (9) ad hoc (2)
**Training coordinators**: specialists (17); non-health graduates (7)	All (24)	training coordination (24) general programme coordination (3)	by specialists	ongoing support (3)
**Specialists:** specialist MDTs (5); in-house psychiatrist (9); no psychiatrist (10) (have psychologist/ PSW in 8 of these)		clinical work/outreach (MDTs) train PC doctors (psychiatrists), LHWs or community members (psychiatrist or psychologists/PSW) lead campaigns (1 psychologist, 1 psychiatrist) lead organisation (4 psychiatrists, 4 psychologists/PSW)	none	

AIIMS: All India Institute of Medical Sciences; anganwadi: maternal and child health government LHW; GASS: Grameena Abhyudaya Seva Samasthe; NBJK: Nav Bharat Jagrath Kendra; RFS: Richmond Fellowship Society; TTK: T.T K Ranganathan Clinical Research Foundation.

#### Heterogeneous human resources

PHWs trained to identify and refer most MNS disorders included doctors, LHWs and/or community members. Decision on which community members were trained depended on the programme’s focus. For example, police officers were trained by organisations that focused on substance abuse (TTK) and suicide prevention (Sneha) (Table 4, S3 Table).

### The community outreach model

#### A novel specialised model

**Fig 9:**
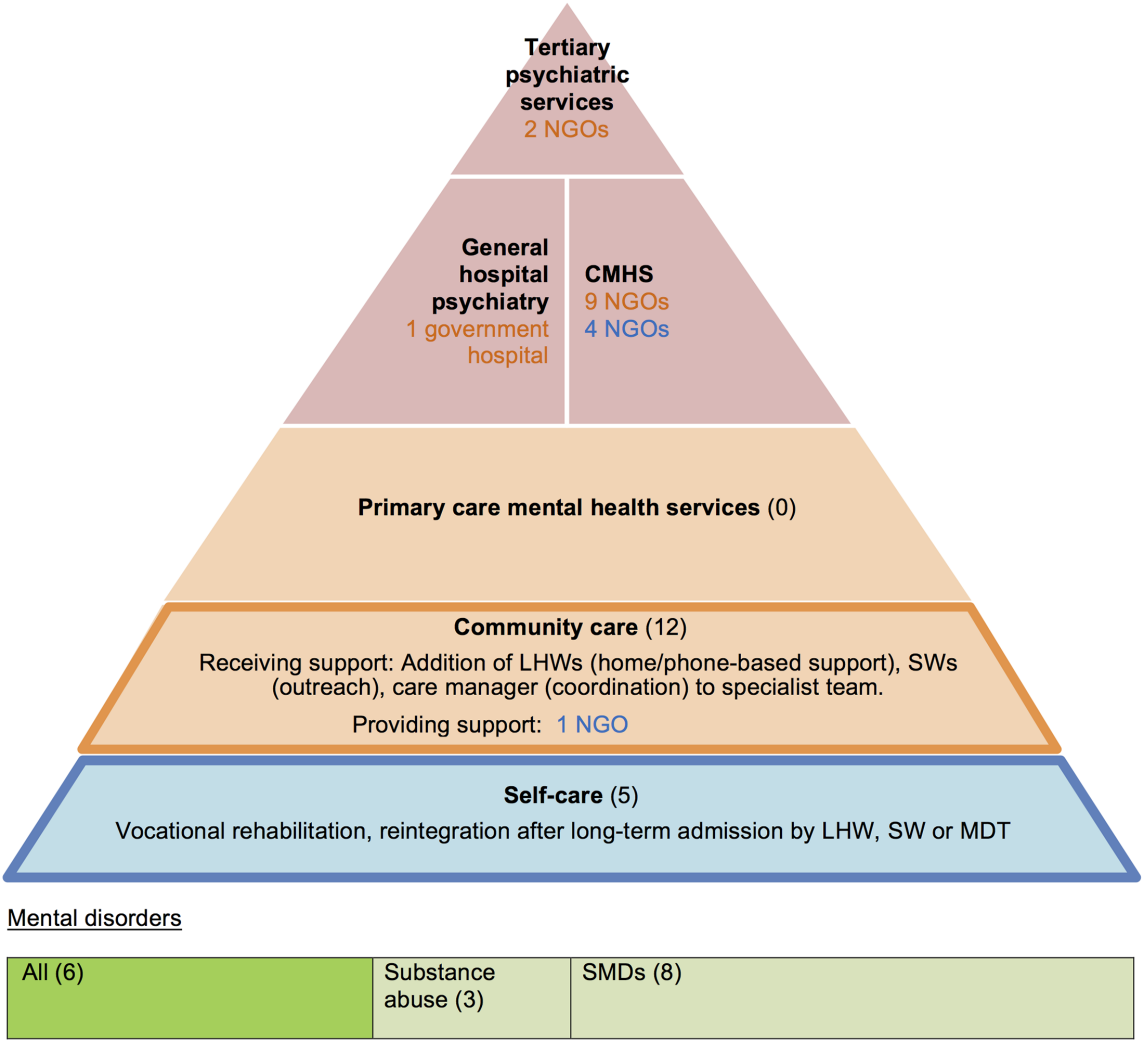
Specialist outreach model characteristics and relationships (n = 17). The colour of the font represents which non-specialist level (primary-, community- or self-care) the organisation supports. CMHS: community mental health service; ANM: Auxiliary-Nurse Midwife; SW: social worker; LHW: Lay health worker; NGO (non-governmental organisation); MDT: multidisciplinary team of specialists and non-specialists; SMD: severe mental disorders.

In this new model, specialist NGOs recruited and trained community-level PHWs to broaden detection particularly of severe mental- and substance use- disorders, and improve their management and continuity of care (Fig 9). As opposed to fully integrated models where specialist and PC services share care, decision-making and location [14], programmes were mostly specialist-centric: psychiatrists assessed patients in outreach clinics, formulated a plan and delegated PHW-led follow-up care (matched care). Some programmes also used PHWs to provide rehabilitative services (including vocational training and supportive employment) (Box 2 example 1; S4 Table). They also completely bypassed primary care centres (Fig 9). Only six programmes differed where PHWs initiated care (stepped care): 1/ LHW-initiated counselling (first step of stepped care) (Saarthak, VOLCOMH (Volunteers for Community Mental Health) (Box 2 example 2); 2/ triage by PHWs to appropriate specialist care or social support (the Banyan urban mental health programme) and 3/ PHW-led helplines (Box 2 example 3).

**Box 2. Community outreach examples**

**Example 1: a PHW-delivered rehabilitation programme—The Chellamuthu Trust vocational rehabilitation units for the mentally ill (Tamil Nadu)**

Psychiatrists refer suitable patients to this residential holistic service which includes vocational training (the five centres offer different options e.g. tailoring, agricultural micro-enterprise) delivered by recovered patients and LHWs, and specialist-led therapies and clinical review. The psychiatrists coordinate and supervise trainers and review patients when required. Chellamuthu Trust run these rehabilitation programmes in addition to community PC programmes (identification and referral model), and specialised programmes for people with mental disabilities.

**Example 2: a remote home-based stepped care mental health programme—The VOLCOMH outreach programme (Mizoram)**

LHWs (recovered users and graduate outreach workers who supervise the latter) who have some roles in the existing substance abuse/HIV programme have been trained to identify mental disorders in the community, provide some psychosocial support (livelihood/benefits) and counselling prior to referring to a clinical psychologist (stepped care). They have no psychiatrist attached to their programme but can refer to a government psychiatrist. The stepped care approach was more feasible for them in their low-resource setting with very limited specialist support.

**Example 3: an urban-based PHW-delivered helpline within Mukthangan Mitra substance abuse hospital (Maharashtra)**

Volunteers (of any background from basic education to professionals) receive one month training and ongoing support/training. They answer crisis calls from people using addictive substances and provide one-off support (as opposed to stepped/matched care). They provide advice, support, counselling (some specifically trained in cognitive- or rational-emotive behaviour therapy). Volunteers receive supervision from a care manager (MH paraprofessional). A hospital psychiatrist supervises and trains care managers.

Similar to collaborative care programmes, these outreach programmes were well funded by multiple donors and income from wealthier patients. Consequently their drug supply was adequate, and some had also built clinical information systems.

#### Greater reliance on specialist human resources

Similarly to collaborative care, community outreach models had one or several well supervised care managers who were experienced PHWs (12 programmes) or specialists (11 programmes) (Table 5). Coordination roles (clinical and administrative) were also divided differently depending on the staff mix in that organisation.

**Table 5:** Specialist outreach model human resources.

Health workers and backgrounds	Nb of pro-grammes	Roles	Training and supervision
**Generalist doctor**	1 (Bapu Trust)	exclude organic disorders (hired just for outreach clinics)	none
**Non-physician professionals**: social worker (SW) (3); nurse/ pharmacist (1)	3	part of outreach teams	by specialists: regular supervision
**LHWs**: primary/secondary school (10); recovered users (5); graduates (3)	15	identification/ referral psychosocial support counselling (5) emotional first aid (3 helplines) vocational training (5) administrative roles (2)	well supported by care managers and specialists
**Community members**: volunteer (any educational level)	1 (Banyan reintegration programme)	reintegrate patients into families/ community	by specialists
Care managers*: experienced LHWs (4); SWs/graduates (8); psychologists (2), PSWs (2), psychiatrists (6)	17	in addition to own clinical roles: patient-LHW-specialist liaison (9) train (4), coordinate (6) and supervise (all) LHWs reintegration activities (1)	regular by specialists
**Administrative coordinator**: graduate	1 (MHAT)	coordinate clinic/homecare activities monitor/coordinate nurses/LHWs	training: intensive palliative course supervision: by head/psychiatrist
**Specialists** (team structure): specialist MDTs (8); in-house psychiatrist (6); no psychiatrist (2) (have psychologist)		outreach clinics (matched care) (13), home visits (1) training PHWs (4) support/supervise care managers/ coordinators (13) and PHWs directly (8). Hierarchical supervision (4) care coordination (see above). trial monitoring, advocacy (SCARF) lead organisation	none

* Some programmes had more than one care manager

MHAT: Mental Health Action Trust; SCARF: Schizophrenia Research Foundation.

Most programmes used LHWs. Within outreach programmes, LHWs identified, referred and provided support. LHWs often performed single interventions to which psychiatrists referred patients such as an anti-stigma trial intervention (SCARF was a selected site) or vocational skills training (Box 2 example 1). LHWs (of any background) also had counselling or first aid roles, particularly in programmes specialised in substance abuse and schizophrenia (Box 2, example 3). Social workers, nurses and pharmacists were used less intensively and only during outreach work to support the psychiatrists work. Only one programme hired a generalist doctor to exclude organic disorders (Table 5).

Their system relied more heavily on specialists than collaborative care models for coordination though their clinical, supervisory and training roles were similar. However PHWs (apart from PC doctors) received more intensive (usually weekly or monthly) support than those within collaborative care programmes, delivered by specialists and non-specialist care managers. Their specialist-delivered training was also longer and ongoing training more frequent than in collaborative care models (S4 Table).

## Discussion

Below we discuss how Indian programmes fit in with the current evidence of effectiveness of models and types of human resources as well as the implications for policy and practice.

### Are the models used appropriate?

**Fig 10:**
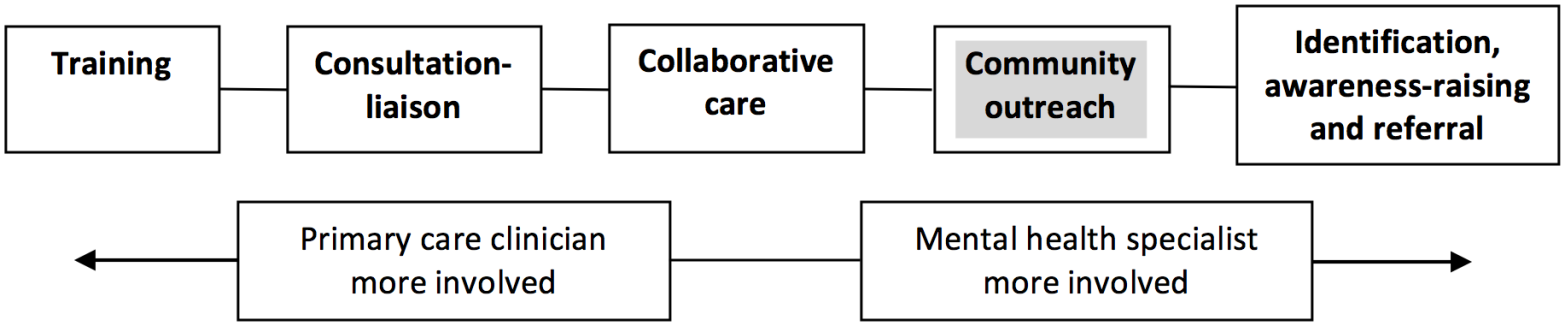
Revised models of primary mental healthcare provision.

Several models of primary mental healthcare delivery in India were different to those described in HICs (apart from the training model). The most prominent finding is that the Bower framework does not fully reflect the models of care in India. Firstly, the consultation-liaison model was absent. Secondly, our study contributed the addition of a fifth model, the community outreach model, where specialist organisations provide a parallel primary mental healthcare services in response to a large unmet need for treatment and support mainly for those are severely mentally ill and their carers [10]. This pattern is known to other LMICs [6, 27] but does not currently figure within existing frameworks of primary mental healthcare. This study’s key finding, and thus contribution to the evidence base, suggests the need for a modified Bower framework to incorporate this new model (Fig 10). However barriers to recommending this new model for scaling up include:

- its heavy reliance on specialists (only possible because of NGOs’ donor funding and some dedicated specialists’ volunteerism) [28];
- its neglect of general physical healthcare;
- no evaluation of its effectiveness and cost-effectiveness, except for one recent trial suggested a modest effect of the SCARF anti-stigma intervention in reducing symptoms and disability from schizophrenia [29]. This is partly because routine data collection and its systematic analysis are poor. Most therapies/ interventions provided in these programmes were not specific or evidence-based as compared to trial interventions [6];
- the attrition of PHWs: contributing factors include reliance on LHWs’ volunteerism, lack of incentives, mentorship, career opportunities and workplace conditions, a phenomenon common to other LMICs [2, 10, 11].

Given that the collaborative care model has most evidence for effectiveness in HICs [30], still less than a quarter of programmes implemented this model. These models differed to those described in the literature which involve PC staff [6, 31], as numerous NGOs bypassed primary care and PC doctors. These programmes worked instead with LHWs, multiple tiers of coordinators and community-based organisations. This discrepancy between ground-driven activities and research-led interventions suggest it would be worthwhile assessing these different cadres’ and community settings’ effectiveness both in India and in other LMICs. In African countries for example some programmes incorporate traditional healers into their collaborative team [32].

Furthermore, the matched care approach (psychiatrists diagnose first then match care to primary care) contrasts to the stepped care approach which features in most of the current evidence base in HICs (i.e. PC staff diagnose and treat, and later involve specialists according to need). Stepped care is less specialist-resource-intensive than matched care and yet is used less in India. This is a feature common to other middle income countries such as South Africa [31]. Furthermore most programmes covered all mental disorders rather than focussing on one disorder (such as depression or schizophrenia). The rationale for these alternative approaches to collaborative care and their comparative cost-effectiveness need to be evaluated. Indeed a large consortium is underway to study collaborative care models for multiple MNS disorders in LMICs [31].

We found that the training model, for which there is little evidence of effectiveness on its own [12], was utilised by a quarter of programmes. However it has remained the Indian government’s DMHP model to train PC staff. This model remains attractive because it is cheap (short training duration), and involves less work for overstretched specialists [12]. However these case studies confirmed the poor sustainability of these models (half the PC doctor training programmes had shut down) due to weak PC systems, drug shortages or perceived ineffectiveness.

Many programmes trained PHWs to identify, sensitise and refer, where specialists retained responsibility for care. This model has some evidence for improving patient and service outcomes in HICs when associated with specialist support [33]. However in India, few programmes provided a support structure and therefore may be as ineffective as the training models.

Though no active consultation-liaison models were identified in India, these are retained in this framework as they continue to be used in other countries and were used in prior phases of two programmes. The evidence is sparse on this model’s effectiveness for depression but it may be useful for other conditions such as substance abuse [11].

### Are the human resources used appropriate?

Many variations of health workforce cadres were identified. We discuss these in light of current evidence for their use.

#### PHWs

Though collaborative care examples in the literature utilise professional PC staff (doctors, nurses, pharmacists, social workers) and graduate care managers [11], few Indian programmes utilise PC doctors (apart from the DMHP sites). In India the PC doctor may not be the most appropriate main primary mental healthcare provider. They may be poorly equipped to diagnose and treat mental disorders because of a/ inherent primary health system weaknesses such as poor medical training, quality of cadre and staff motivation and b/ insufficient mental health training [10]. Some states such as Tamil Nadu have addressed these weaknesses by restricting PC doctors’ roles to identification and referral.

Many Indian programmes substituted a lay for a professional workforce as this was more feasible given the dearth of specialist and professional human resources [2]. For example LHWs provided psychosocial support and adapted psychotherapeutic interventions rather than social workers or psychologists. Furthermore, LHWs were more retainable and better placed to identify mental illnesses given they were usually stable community residents.

Most noticeable in their absence was the dearth of Indian nurses utilised in mental healthcare. They have maintained traditional roles (e.g. providing first aid and injections) unlike in African countries where nurses are more abundant and have greater task-sharing roles (MNS diagnoses, symptom management and repeat prescriptions) [15, 34]. Cross-learning of task-sharing experiences between LMICs may provide further currently unexplored opportunities for India.

#### Care coordination

Professional or specialist care managers improve primary-specialist mental health collaborations [18]. Within Indian collaborative care and outreach models they were less specialised and had more tiers of coordinators than in study settings [6]. However they received as much supervision which is likely to improve confidence, detection, treatment and treatment adherence [2]. Their roles were also broader to further minimise the need for specialists’ involvement. These different forms of care coordination and of their support need further evaluation.

#### Specialist support

Specialists were used differently at PC level in India compared with HICs. Particularly in better resourced settings (NGOs or wealthier states) psychiatrists had greater responsibility (outreach clinics and matching care, leading programmes) in addition to training and supporting PHWs. In poorly resourced settings the opposite occurred: specialists were not available or had been phased out. This difference in specialist availability between better- and lower- resourced settings in India is also reflected across LMICs whereby low-income countries (such as Ethiopia, Uganda and Nepal) tend to not have the resources for mental specialist involvement in primary care whereas middle-income countries (such as India and South Africa) do [31]. Given the PC system weaknesses additional help formulating initial diagnoses and a management plan may be relevant [6, 10]. However, the dearth of specialists may inhibit their scalability at primary care level.

Furthermore few specialists are willing to take on more managerial or supervisory roles for community care. This may be explained by their attitude: some specialists disregard task-sharing as they believe PHWs’ limited training is insufficient to provide adequate care [10]. They may also feel uncomfortable with PHW supervisory roles as they are not trained or incentivised to provide these.

### Study limitations

Projects may change and we are aware of many country-wide developments since 2012 (e.g. new CMHP projects in ANT/Ashadeep, reopened/ restructured PC doctor training (Manasa)) which have not been captured by our study. This study’s methods did not allow for impact and process evaluation such as evaluation of costs/resources used, and also did not assess children services or private for-profit sector delivery. These should feature as future research priorities.

Web-searches and snowballing may have biased towards better resourced programmes, though five of our programmes were remote, basic programmes with no websites (identified through local snowballing). Sampling of participants within programmes was also subjected to convenience sampling as they were chosen by organisations based on our stipulations. It is possible we were presented only with the best staff or those who would portray the organisation’s work positively. However, we used multiple forms of data, interviewer cross checking, and return visits (two case studies) to minimise this risk.

Multiple case study analysis can result in disaggregated results. The authors attempted to maintain the integrity of all 72 programmes during data-aggregation through the use of pattern recognition and matrix analysis [25]. Individual programme details are available in S1–S4 Tables.

### Implications for practice

This study identified important innovative models and human resource use which may have potential for profound change if implemented at scale. For example the DMHP’s programme implemented via government PC is using an ineffective training model. It may need to reconsider its PC delivery model. Its targets have also not been met due to political and health system weaknesses [10]. Health system strengthening through the National Rural Health Mission (NRHM) and the broader vision of universal health coverage [3] will be necessary to improve India’s PC delivery and quality. This would then support multifaceted primary mental healthcare delivery. Opportunities to incorporate or collaborate with innovative NGO initiatives described above may help this process. Exploring collaborations with for-profit private care (70% of healthcare in India) should also be explored though many in India and other LMICs would resist partnering with the private sector until it is more regulated and accountable [3].

Within a country as diverse as India, it is unlikely one model would suit the whole country nor every MNS disorder. Recommendations to scaling-up concepts are more appropriate and would allow models to be adapted to local needs and resources. For example community outreach models may be appropriate when targeted to severe mental disorders. With regards to the workforce, these findings support the current evidence and policy analysis that the add-on of a care manager and use of LHWs to provide psychosocial support are crucial to effective and accessible primary mental healthcare [6]. They seem more acceptable and feasible than PC doctors at identifying and treating cases, and at care coordination and at linking with specialists. This expansion of roles and of PHW workforce correlates with policy recommendations [35] and also seems acceptable to health workers [36].

However this study also highlights the importance of ongoing specialist support. While the Indian mental healthcare policy is a great step towards formalising universal mental health coverage, the success of these recommendations relies on their implementation and on the buy-in and redistribution of specialists [2, 10]. Given the large geographical variations in availability of specialist resources within India, greater use of mobile technology needs exploring. Using mobile phones for supervision has facilitated specialist-care manager communication (explored by many NGOs), as has telemedicine for diagnosis/follow-up [37].

## Supporting information

S1 Text Data collection tools for case studies. (DOCX)

S2 Text Summary sheet to be completed after interviews/visits. (DOCX)

S1 Table Characteristics of collaborative care programmes. (DOCX)

S2 Table Characteristics of training programmes. (DOCX)

S3 Table Characteristics of identification, referral and sensitisation programmes. (DOCX)

S4 Table Characteristics of community outreach programmes. (DOCX)
