# Supplementary material for: Human resources and models of mental healthcare integration into primary and community care in India: Case studies of 72 programmes
Source: PLoS One. 2017 Jun 5;12(6):e0178954. doi: 10.1371/journal.pone.0178954 (PMC5459474; doi:10.1371/journal.pone.0178954)
Supplement: S1 Text — (DOCX) [file pone.0178954.s001.docx]

S1 Text. Data collection tools for case studies.

- 1. Interview guide for semi-structured interviews

| **Category** | **Content** | **Interview Guide** | **Prompts** |
| --- | --- | --- | --- |
| **Questions to all staff (founders/ managers/ coordinators/ NSHWs) about the programme and various roles** | 1)Program description    2) Role description of non specialised workers  3) Specialist/ supervisory staff Qualifications, expertise and roles | 1a). Why and what the programme is for?  1b). Major achievements and milestones since the program began  2a) The role of NSHWs within the mental health program?  2b) Other NSHW roles  2c) Who are the NSHWs?   - Titles - Their roles - How MH roles fit in non-MH roles? Challenges to this. - Their workload   2d) Do you have any role as supervisor/coordinator toward NSHWs ?  3a)Founder/manager/ coordinator’s expertise/background in delivering mental health  3b)NSHW expertise in delivering mental health  (Ongoing expertise and oversight in the area of mental health services)  3c). What supervisory roles to the coordinator / roles of specialists(MH specialists) ? | - Why the program started/was founded? Philosophy of the program? What personal reason for setting up a program? - Can you share examples of what issues have come up over time that may have changed the direction of your programme or refined your current work? - have you helped designing practices which respond to mental health concerns? Examples to illustrate. - And also review available documents   - Prompts: NSHW roles in detecting, treating mental disorders, follow up, training of other NSHWs   - Prompts: roles in advocacy, livelihood programmes   - Who supports NSHWs in their MH work?   - what is your background/ training?   - What training do the NSHW’s /specialists get? (the Length of training and if it’s repeated)   - experience of the person who provides this expertise for the program   - What roles: support, supervision, training, overseeing |
| **Specific questions if the staff are recovered patients /users) | 1)**Staff (who are recovered patients) views  2) their contribution to the program me | 1. clients view about their work and mental health which relates to their status as recovered users 2. any specific roles they can have related to them being a recovered user | - 1a. Do their patients accept them as recovered patients and also as staff? - 1b. Give examples of some of your strengths and weaknesses as a recovered user in your work. |
| **Specific questions for religious leaders |  | Specific questions relating to the religious element | - 1)Did any client oppose you saying that you are not specialised. - Any circumstance where the client s were unhappy about the service - What are their linkages to the allopathic system? |
|  | 1) Is the service affordable and is it accessible to patients?  2)Socio-Demographic details  3)Service characteristics | 1a.) How much does it cost to get from door to door  1b)How often do they need to come to clinic  1c) How far is the clinic  1d) Other indirect costs to the patients  2a) What category of people come to them and their income level  2b) What are their reasons for attending  3a) What are the most prevalent disorders they see (& presentations  3b) Infrastructure of the MH service   - Capacity of the clinic - Drug supply (from where, cost, reliablility/stock)   3c. Linkages to   - DMHP/NRHM - Other MH services/ organisations   Traditional/religious healers | - Are they (clients ) able to reach the service place - Are there situations where the programme is running in one place and the service is accessed dominantly in number by other village people - Are they(clients) able to afford to come and take the mental health care service |
| Interventions by NSHWs and by others (see how it links with specialist interventions; e.g.: psychiatric nurses/social workers/ psychiatrists)  Other interventions | 1)Addressing mental health concerns  2)What are the other livelihood program for the community ( patients family) with the mental health programme | 1a) Identifying the mental health concerns/screening  1b) Awareness raising and Screening  1c) Treatment- Which by national (NSHWs)  1d) Follow up  And supervision  2a) SHG self help groups  2b) Income generating activities  2c) Any other groups/ interventions | - What role do you play in the program's efforts to conduct screening to identify mental sickness - What treatments doe NSHWs perform? (prompts: prescribing, counselling, motivational interviewing etc) - How do you coordinate services to ensure that mental health needs are communicated, and that follow-up occurs? - Can you share an example of a referral made this year and its resolution? - What follow-up is done by NSHWs? By which NSHWs? |
| Monitoring | Monitoring | 1. Ongoing monitoring and overview of the programme | - What do you do if you detect problems or weaknesses with the programme's mental health services? - How do you monitor delivery of the programme's mental health services and the programme's compliance with regulations? - Have you requested formally patient feedback |
| Success or limitations of implementation | Implementing Mental Health Services/impact | 1)Success and limitations in regard to NSHWs   - Recruiting NSHWs - Training/Ongoing training - Supervision - Quality of their work - Retaining   2)Community Involvement | - What challenges of using NSHWs = examples of situations - Any of the Project challenge or opportunities like issues of funding, acceptability for NSHW, - Acceptability for NSHW community users - Supervision/support from specialists at government/local policies - How often do you visit each setting? How is the schedule implemented across all program options - How is your work /competency evaluated - How are you rewarded for your involvement - How are NSHW involved in clients (patients), family (community), information, observations, and concerns about the mental health? |
|  | Future of Mental Health Programme | 1. Your opinion on the future of mental health program you are working in and the future roles as non specialised health care workers | - Do you think this program will sustain with non specialised care workers + why? - Any recommendation you had given for the programme to make it more nice/ improve your own programme. |

- 1. General observations during site visits

**Domain 3: environment**

1. What is the physical setting like? Is it adequate for what it is trying to do? Are things functional?

**Domain 4: Health system in which the programme functions (ie try to go an meet some of the other health systems in that area eg private providers, healers, pharmacists, other PHCs)**

1. What MH and general health services and alternative services are close by?
2. How does the mental health service function within the PHC system?

**Domain 7: program resources**

1. Are there any visible discrepancy of resources around the clinic? (human, financial, transportation, other)

**Domain 14: accessibility of services**

1. Is the location central, is the provision of transportation accessible for patients? Is there any pharmacy close by?
2. What are your opening and closing hours?
3. What in-home/outreach services are provided?
   1. Documentary analysis (if available)

**RECORDS ANALYSIS**

**Administrative records and other literature on the clinic (meeting notes, reports, evaluations etc):-**

1. Local language and terminology to describe phenomena
2. **Domains 1 and 2: History of the program and key events**: when the program was established, where, why, what, who and how. Timeline and major achivements
3. **Domains 3 and 14: environment** **in which the program functions and accessibility of services**
   1. figures of the locality (population covered socio-economic groups, patient characteristics, number of people from different backgrounds, ages, sex).
   2. Details of the locality of Gumballi
   3. Details of the infrastructure of the building and programme
   4. Affordable fees, service hours, in home/outreach services? Do the plans (in written proposals and recommendations) match the current status of the clinic?
4. **Domains 4 and 6: health system and broader systems:**  What information can we find from reports or internal documents and wider reading (perhaps ask at district level) about the existing health system and the programme’s engagement with broader systems (political, social systems, advocacy etc)
5. **Domain 5: programme conceptual framework**
   1. What is documented as the programme’s conceptual framework and orientation of services.
   2. For evaluation: what are their indicators? Just service indicators/monitoring of activities or also quality indicators (how is this changing patient outcomes, improvement in patient care, improvement in accessing the right populations etc)? Have there been any previous evaluations?
6. **Domain 7: Program resources:**
   1. facts and figures on types/quantity/quality of resources (human, financial, transportation, other) and change over time (increase, decrease, changes of resources etc)
7. **Domain 8: project management**
   1. Look to collect and photocopy all staff job profiles and training manuals or courses to compare to their actual work.
   2. See if we can have access to the budget or financial statements in reports to look at financial stability of the project
8. **Domains 9 and 10: pathways to care, referrals and client populations**
   1. Any reports, publications from that project on pathways to care, help-seeking, referral networks etc.
   2. Administrative records for sociodemographic characteristics,
9. **Domains 11, 12, 13 and 13b: interventions and medicines**
   1. Any reports/evaluations with outcomes analysis of interventions from records or studies.
   2. Any records about medication supply and usage
10. **Domains 14: Accessibility of services**
    1. Any reports or feasibility studies or evaluations that look at distance and cost (geographical accessibility and affordability)
