# Supplementary material for: Human resources and models of mental healthcare integration into primary and community care in India: Case studies of 72 programmes
Source: PLoS One. 2017 Jun 5;12(6):e0178954. doi: 10.1371/journal.pone.0178954 (PMC5459474; doi:10.1371/journal.pone.0178954)
Supplement: S2 Text — (DOCX) [file pone.0178954.s002.docx]

S2 Text. Summary sheet to be completed after interviews/visits.

| To be completed after the visit/IDI or to summarise a transcript/audiorecording. Give a summary of:   - recorded interactions - off-the-record interactions - observed information - potential insights from the above   Put in relevant quotes (either from memory of event or copy and paste from transcript) |
| --- |

1. **Date interviewed:**
2. **Name of programme/project**
3. **Name(s) and designation(s) of person/people interviewed/visited**

| **Name(s)** | **Designation/other relevant details** | **Recorded interview (Y or N)** |
| --- | --- | --- |
|  |  |  |
|  |  |  |

1. **General observations and mood of the interview**
2. **Programme description** (Brief history of the programme, what is the model of mental health care delivery, and how may it fit in with other concurrent programmes, what are the costs/affordability of the service, what is the service infrastructure like, monitoring of the program)
3. **What are the characteristics of the population/patient population served** (sociodemographic details of population, types of mental disorders treated/detected etc,) **and their behaviours** (pathways to care, utilisation of the service, reasons for attending)
4. **Who are the NSHWs? What are their qualifications/training? job titles? Expertise? How are they selected? What are their incentives/pay? Are they motivated to stay in their work and why(not)? (also mention issues like transport, accessibility, their availability etc)**
5. **Describe the roles/responsibilities of NSHWs including whose views are represented (founder, manager, coordinator, specialist, NSHW). Are they motivated to do the work (eg are they salaried or not, other incentives)? Include issues like case load (amount of patients they see and what types of problems), work load (how they feel about their workload and the burden of what they are doing).**
6. **Describe the roles of any specialist or supporting staff to NSHWs. (and their qualifications and expertise). Again mention whose views are represented**
7. **What linkages does the NSHW service delivery have to other sectors?** (government programme/DMHP, other private/civil society programmes, the spiritual/ healing sector). **Give details.**
8. **Interventions done by NSHWs** (case finding/identification, awareness raising, treatment (pharmacological and non-pharmacological), referral follow-up)
9. **Wider involvement of NSHWs: eg. Participating in programme decisions** (how hare mental health needs communicated? Do they have any authority in the direction of the programme?) **or their community involvement** (social programmes, SHGs, in providing information, advocacy etc)
10. **What have the successes and limitations been of the programme? Particularly with regards to NSHWs. What solutions or recommendations do they have for improvement of the programme, or of the involvement of NSHWs.**
11. **Their opinion on the future of the mental health programme, and any vision of scaling-up their model**
12. **Other relevant details (e.g. issues seen during visits – refer to data collection tools)**
