# Supplementary material for: Human resources and models of mental healthcare integration into primary and community care in India: Case studies of 72 programmes
Source: PLoS One. 2017 Jun 5;12(6):e0178954. doi: 10.1371/journal.pone.0178954 (PMC5459474; doi:10.1371/journal.pone.0178954)
Supplement: S1 Table — (DOCX) [file pone.0178954.s003.docx]

S1 Table. Characteristics of collaborative care programmes.

| **Programme** | | **State** | **Loc-**  **ation** | **Length of programme** | **Mental disorders (MD)** | **Specialist platform** | **PHW platform** | **Level of PC/community and specialist collaboration** | **Stepped/matched care** | **PHWs: background and roles** | **Specialists: background and roles** | **Care manager: background + roles** | **Other care coordinator: background + roles** | **Training + supervision of care manager/ coordinator** |
| --- | --- | --- | --- | --- | --- | --- | --- | --- | --- | --- | --- | --- | --- | --- |
|  | Collaborative care with primary care (PC) + community care | | | | | | | | | | | | | |
| **Banyan-Rural Mental Health Programme (RMHP)** | | Tamil Nadu | Rural (R) | Banyan set up 1993; RMHP: 2004- now | all | CMHS (NGO) | PC+ community | Intensive contact for LHWs (weekly or fortnightly contact with specialist and daily contact with coordinator). Minimal collaboration of PC doctor with specialists (currently trying to improve). | Matched care determined by psychiatrist (? Combined with some elements of stepped care re homeless/non clinical issues) | CHWs (LHWs): awareness, detection, follow-up, psychosocial support/ coping strategies/ counselling (home). Intensive apprenticeship and periodic training with psychiatrist+ coordinator.  Social worker (SW): joint visits with CHW, psychosocial assessment and care (stigma, livelihood, benefits etc), intervention planning/facilitations  Generalist doctor: medical role only. | Psychiatrist: diagnosis, treatment. Supervise care manager Psychologist: therapies. (outreach clinics in PC). | Coordinator (Postgraduate): joint/separate home visits with CHWs. Liaise between patients, CHWs and specialists (CHWs liaise with patients). |  | Periodic training and ongoing support supervision by psychiatrist - regular meetings. |
| **Chellamuthu Trust-Community Mental Health Programme (CMHP)/ Sivakasi** | | Tamil Nadu | R | Chellamuthu established 1992. CMHP: 2000- now | all | CMHS (NGO) | PC+ community+ self-care | Close collaboration between specialists and LHWs (both NGO-linked). Minimal collaboration with PC (used as a platform for delivery). | Matched care determined by psychiatrist. | LHWs: identification, referral, follow up, home-based care, contribute to income-generating activities, awareness raising, surveys (home visits). ). Initiation of Self health groups (SHGs) Other health workers: screen children for disabilities. PC staff: identify and refer (also have general roles. Social workers: coordination, outreach team, supervise LHWs. | Specialist team (psychiatrist, psychologist, PSW): diagnosis, treatment, follow up (outreach clinics in PC, +early identification camps for child disorders). Psychiatrists trained PHWs. | Community care workers (community volunteers) (LHWs): care roles and Liaise between patients, PC and specialists. | Project coordinator (social worker background) : administration of project and supervises social workers. | LHW supervision by social workers (SW) (coordinators/ part of the outreach team). Some shared home care and SW activities. Trained by psychiatrists. |
| **CHAD (department of community health, Christian Medical College, Vellore)-CMHP** | | Tamil Nadu | R | CHAD set up 1974. MH component integrated 1990s | all- mainly depression detected | General hospital (NGO) | PC + community | Close collaboration between specialists and LHWs (NGO-linked, and government ANMs). Minimal collaboration with PC. | Matched care determined by psychiatrist. | Health aides (LHWs): generalist LHWs (focus ANC/PNC) with mental health roles: identification, referral, some psychosocial support; volunteers (LHWs): follow-up, some are lay counsellors (main MH focus);  PC doctor: identification, referral, records/data collection; ANM: identification/ referral. Social workers: most medical social workers with MH roles | 2 psychiatr-ists: diagnosis, treatment, follow-up, rehabilitation (outreach clinics). Used to train LHWs. Psychiatric social workers (PSW) also provide counselling | Health aides (LHW): identification, referral and psychosocial support. Liaise between PC, patients, social workers and specialist team. | Project coordinator : administration of project and supervises social workers. | LHW supervision by social worker -used to be every month, now once a year. Follow up and supervision on community visits. Other roles: counselling, awareness raising and monitoring |
| **GASS (Grameena Abhyudaya Seva Samasthe)_ Community based rehabilitation (CBR) workers** | | Karnataka | R | 1996 GASS; 2000: MH programme | all | CMHS (NGO - Basic Needs UK); Private (for profit) psychiatrist | Community (NGO - GASS) (+ PC community ANMs) | Minimal collaboration with specialist. Good collaboration between NGOs and PC staff. | Matched care by psychiatrist | Community-based rehabilitation (CBR) workers: identification, referral, follow-up, some counselling, psychoeducation, awareness, support, bring patients to camp (also disability roles). Government ANMs (LHW): identification, referral. (home visits). ANMs trained by BasicNeeds-UK and GASS, supervised by MH coordinator. | Visiting external private psychiatrist: diagnosis/treatment camps. | Community based rehabilitation (CBR) worker: clinical roles as under 'PHW'. Liaise between community and GASS team (but no links with specialists). |  | CBR workers used to be trained by psychiatrists when they were more involved. Supervised by MH coordinator, mental retardation specialists, physiotherapists. BasicNeeds-UK provide technical support.  MH coordinator supervised by GASS head. |
| **T.T.K. Ranganathan Trust Clinical Research Foundation (TTK) (NGO)-rural camps with local organisation (CBO) partnership (outreach clinic with PHW support)** | | Tamil Nadu | R | 1980 (TTK established). Rural camps since 1989- now | Alcohol abuse | Specialist hospital (NGO) | Community (CBO) | Regular collaboration between specialist NGO, CBO and PC doctor with aim to make CBO independent. | Matched care - triage by social worker | CBO animators (LHW): identify, psychosocial support, bring people to camp, follow-up, raising awareness. Clinic volunteers (LHW): support to team only (Home/community care).  PC doctor: organic disorder exclusion. Contributes to (during camps) and later independently provides alcohol dependence assessment, treatment and follow up and if relevant referral. | Specialist team from NGO (psychologists, PSW) outreach clinics every 2 months. No psychiatrist: assessment, detoxification and follow-up. Also train and supervise animators. (planned specialist withdrawal) | CBO animator (usually a graduate with no health/MH background): Roles as under 'PHW'. Liaises between CBO, PC doc and TTK. |  | LHWs and PC doctors trained and supervised by PSW and psychologists (NGO) during camps. |
| **Karuna Trust - Gumballi (early programme) (in-depth case study)** | | Karnataka | R | Karuna Trust/VGKK since 1987. MH component since 1991. PHC GUmballi since 1996 | all | Specialist hospital (government-NIMHANS) and community (NGO) | PC+ community | Close collaboration between PC doctor, NGO and psychiatrists (government hospital). Intensive bedside-training model of consultation-liaison. (the programme has since moved to simply training PC doctors with minimal ongoing support from specialists). | Matched care determined by psychiatrist (stepped care in later programme - see educated and training). | PC doctor: refer patient to camp, sit in with psychiatrist, follow-up after treatment initiation. Community-based LHWs (multipurpose workers): minimal training in mental health to identify and refer. | Psychiatrists: fortnightly clinics in PC. Supervised PC doctor. (planned specialist withdrawal) |  | PC coordinator: (graduate) manages all aspects of PC. PC doctor: liaises between patients and specialists. | Significant experiential teaching, supervision and support from psychiatrists. |
| **MICP (Malappuram Initiative in Community Psychiatry) - an add-on to the DMHP initiative** | | Kerala | R | 1996-now | all | General hospital (NGO) | PC + community | Ad hoc and minimal collaboration between psychiatrist (NGO/hospital) and PHWs (government LHWs). | Matched care determined by psychiatrist. | ANM, ASHA, pariraksha nurses (panchayat-level homecare nurse): identify, refer, follow-up including check medication adherence, facilitate rehabilitation activities, (also general roles). (Home visits). Panchayat volunteers, health inspectors: identify and refer to the pariraksha nurse who then sends them to PC. PC doctor: identify, refer and follow up medical dosage changes (PC-based); mainly exclude organic cause (during camps) | Psychiatrist: diagnosis, treatment, follow-up (outreach camps). Also supervises all community-level staff. Psychologist: available to be referred to (even for children). |  | Only by psychiatrist (supervises all community staff and does outreach clinics). | None |
|  | Collaborative care with community care (not PC) | | | | | | | | | | | | | |
| **Ashadeep-outreach programme** | | Assam | R | Ashadeep founded 1996. MH outreach: 2000-now | all (homeless women) (started as just SMDs) | CMHS (NGO) | Community | Moderate collaboration between specialists (in NGO) and LHWs (in CBOs - community based organisations) during outreach clinics. | Stepped care (usually see LHWs first before having access to psychiatrist). | LHWs from CBOs: awareness, psychosocial support. Some do counselling (home visits). CBO social worker: identify and refer homeless people (outreach work), awareness raising. LHWs/SWs also have general health or development roles. NGO generalist doctor: part of camps: before psychiatrists were employed, they used to diagnose, treat, follow-up at outreach camps. Now just physical treatment+care in halfway home. Training initially by Ashadeep, then taken over by CBO. | Psychiatric team employed through NGO (psychiatrist, psychologist): diagnosis, treatment (CBO-based clinics). Psychologist also supervises social worker. Specialists and leaders: clinical and organisation problem solving/consultation clinics for CBOs (monthly Ashadeep-based clinics). | CBO-level manager (experienced LHW) and Ashadeep coordinator (initially only): Liaise between LHWs, patients, specialists and organisations regarding activities, needs and clinical information. Supervise LHWs. |  | Supervised by social worker who is supervised by psychologist. |
| **ANT-outreach programme (Ashadeep-linked CBO)** | | Assam | R | ANT founded 2000. MH outreach set up in 2008 | all | CMHS (NGO) and general hospital psychiatrist | Community (CBO) | Minimal collaboration of CBO with government specialists (only visit for camps). Greater collaboration and support from MH NGO (Ashadeep) | Matched care by psychiatrist | LHWs: identify, refer, awareness, psychosocial support, some administer medicines (home visits). Trained by care manager and directors. Generalist doctor: organisation leader, provides general medical and some mental health care including counselling (PC-like clinic) | Visiting external government psychiatrist (monthly camps): diagnosis and treatment. May access consultation clinics at Ashadeep (monthly). | Experienced LHW (senior LHWs): clinical roles as for PHWs. Liaises between patients, LHWs, ANT and Ashadeep directors if necessary. Also trains LHWs, awareness raising. |  | Supervised by programme director (a general physician). |
| **Ashagram** | | Madhya Pradesh | R | Ashagram founded 1980s. MH programme since 1996 | all | Private (for profit) psychiatrist; CMHS | Community + self help | Moderate collaboration between private psychiatrists and NGO coordinators/LHWs. | Matched care by psychiatrist | Key worker (LHW): identify, community follow-up, adherence, surveys, awareness raising. Bring patients to clinic to doctor or psychiatrist outreach clinic; community self help groups: general support;  BAMS doctor: follows up and monitors patients after psychiatrist management initiation. Relies on key workers to send him patients. Also attends some psychiatrist-led consultations in clinics. | Visiting external private psychiatrist: outreach clinics (used to have a PSW) | Experienced LHW (called 'mental health key workers'): Liaise between LHWs, BAMS doctor, head of organisation and psychiatrists. Clinical care as under PHW roles. LHW training and supervision (used to be done by psychiatrist and supervision by PSW). |  | By coordinator - (experienced key worker) - regular support. Coordinate programme. |
| **Chellamuthu Trust - Sathya Sai treatment camps** | | Tamil Nadu | R | 2001- now | all | CMHS (NGO) | Community (religious organisation) | Minimal collaboration of specialists with Sathya sai volunteers: no organised support structure between specialists and LHWs but communication during camps. PC doctor commissioned punctually to provide service at camps - no ongoing care collaboration. | Matched care (decided by psychiatrist) | Sathya Sai volunteers (LHWs) identify and refer, also follow up including medical adherence and side effects, bring patients to camps;  PC doctors: exclude organic causes, may refer to camp too | psychiatrist, psychologist, social workers: monthly outreach clinics. ad hoc supervision of volunteers. | Sathya Sai volunteers (religious volunteers): liaise between patient, volunteers and specialist. organise camp and mobilise/get patients there. Mobilise/raise funds for these camps. Also do identification, and community follow-up for side effects etc. |  | No organised supervision but ad hoc support during outreach clinics. Two days training by specialist team initially. |
| **Banyan- Family Planning Association partnership** | | Tamil Nadu | Urban (U) | 2010-2012 | all (women health) | CMHS (NGO) | Community care (gynaecology NGO) | Intensive contact and co-consulting between gynaecologist and psychiatrist, with a view to maintaining a consultation-liaison approach | Stepped care: seen by gynaecologist first, referred to psychiatrist clinic if needed. | Gynaecologist: opportunistically diagnoses and treats MDs, and follows up (though still lacks confidence too). Still relies on psychiatrist to confirm diagnoses. Aim to gradually hand over to gynaecologist with referrals when problematic | Psychiatrist: ongoing training of gynaecologist and does weekly outreach clinics. (planned specialist withdrawal) |  | By gynaecologist. Liaise between patients and psychiatrist. | Intensive ongoing training and weekly support by a Banyan psychiatrist. |
| **Ashwini** | | Tamil Nadu | R | Ashwini founded 1987. MH component since 2004 | all | Before: specialist hospital psychiatrist); now: informal friend psychiatrists. | Community (general health hospital NGO) + self help | Moderate collaboration at the beginning with NIMHANS psychiatrist (regular training, clinics, support) with care coordination by gynaecologist. Now no collaboration (no involvement of psychiatrist apart from referring to them) | Matched care by gynaecologist (PHW) | LHWs (volunteers): do identification, awareness, referral, psychoeducation to family and patients;  LHWs (health animators): do the same plus informal counselling, follow-up, help set up self-help groups;  gynaecologist: gets patients referred to her from other hospital docs. Does all the diagnosis/treatment. Also trained LHWs. | External psychiatrists: very minimal involvement. Gynaecologist phones psychiatrist friends if difficulties. (early programme: visiting NIMHANS psychiatrist outreach clinic, then for training gynaecologist, doctors and nurses) |  | Gynaecologist: does all the MH work, referred from health animators, volunteers and .hospital doctors | Used to have regular support from NIMHANS psychiatrist. Now only refers when needed or speak to psychiatrist friend. |
| **SACRED -outreach MH programme** | | Andhra Pradesh | R | SACRED founded 1999. MH (BN partnership) since 2001 | all | General hospital (government district psychiatrist) + CMHS (Basic Needs India (BNI)-NGO) | Community (disability NGO - SACRED) + self help (several CBOs) | Minimal collaboration with specialists, but moderate collaboration between mental health NGO (who helps monitor programme), disability NGO (runs the programme) and CBOs (implement self-care and identification). | Matched care by psychiatrist | Development workers (SACRED LHWs): identify and refer, they do follow up and medication adherence. Also lobby government and lead self help groups. Trained by BNI and NIMHANS.  Caregiver forum at village level (CBOs): self-help support and voice for rights of patients and carers. These are grouped in a larger federation which represents these caregiver groups. Supervised by CBR workers/ coordinators. | External district psychiatrist: available for referrals. |  | Several coordinators who are not psychiatrically trained and no care role:  SACRED CBR coordinators (administrative coordination of LHWs),  SACRED training coordinators (monitor+coordinate training to other CBOs+own development workers);  BNI mental health coordinators (monitor programme) | Most training by BNI coordinators/ heads. Supervision hierarchy (see under care coordination roles). |
| **NBJK (Nav Bharat Jagrath Kendra) /RINPAS-outreach programme (outreach clinic with PHW support** | | **Jharkhand** | R | RINPAS (old hospital since 1795) – MH with NBJK since 2002 (though break between ~2006-2010) | all | specialist hospital (RINPAS) (government) +CMHS (NGO - Basic Needs) | community (disability NGO - NBJK) +self-care (CBOs) | moderate collaboration between NGOs and CBOs. Minimal collaboration with RINPAS specialists | matched care by psychiatrist. | NBJK NGO volunteers (LHWs) - (recovered patients or community members). Identification, referral/facilitate access to DMHP outreach clinics, follow-up, awareness, psychosocial support (Home care)  Clinic volunteers: supportive auxiliary role;  CBO partnership volunteers: livelihood activities, care + psychoeducation for families and awareness raising | External government psychiatrists (RINPAS): monthly outreach clinics at PCs/ district hospitals, receive referrals from NBJK. No supervision/ongoing support to any LHWs. |  | NBJK (NGO) Mental health coordinator (non-healthcare graduate): oversees programme, is link between LHWs and specialists. Also trains and supervises LHWs. | Supervised by NGO (NBJK) programme manager, who is in turn supervised by MH NGO (Basic Needs) coordinator |
