# Supplementary material for: Human resources and models of mental healthcare integration into primary and community care in India: Case studies of 72 programmes
Source: PLoS One. 2017 Jun 5;12(6):e0178954. doi: 10.1371/journal.pone.0178954 (PMC5459474; doi:10.1371/journal.pone.0178954)
Supplement: S2 Table — (DOCX) [file pone.0178954.s004.docx]

S2 Table. Characteristics of training programmes.

| **Programme** | **State** | **Location** | **Length of programme** | **MD** | **Specialist/ support platform** | **PHW platform** | **Form of collaboration** | **Level of specialist/ non-spec-ialist colla-boration** | **Roles and training of PHWs/ community** | **Roles of specialists** | **Training coordination and delivery** | **Training/ supervision for coordinator** |
| --- | --- | --- | --- | --- | --- | --- | --- | --- | --- | --- | --- | --- |
| **Government of India (GOI) DMHP - Chamarajnagar - PC doctor training** | Karnataka | R | DMHP since 1996. Chamarajnagar DMHP PHC work since 2005 | all | Specialist +general hospital (government). | PC | Education and training (E&T) - PC | One-time training only | PC doctor: plan for 3 days x2/year for 5 years (total 30 days planned) but most only trained 1 to 3 batches (ie 3 to 9 days). class based training, with video and some clinical training (patients brought in). content diagnosis, treatment, and educate family | General+ specialist hospital psychiatrists train PC doctors and do clinical work. DMHP psychiatrist post vacant so no support. | By department of health and family welfare joint director (MH). | Director of the department of health and family welfare. |
| **GOI DMHP- Karwar - PC doctor and ANM (auxiliary nurse midwife) training** | Karnataka | R | As above | all | Specialist +general hospital (government). | PC | E&T - PC | In practice, only one way training (as vacant post for DMHP psychiatrist) | As above for PC doctor. ANM training: 1 day to identify, refer, basic support. | As above for PC doctor. ANM training by DMHP team (psychologist, psychiatric nurse and PSW). | As above for doctors, by district DMHP team for ANMs. | As above for doctors, programme officer supervises DMHP team. |
| **GOI DMHP- Shimoga - PC doctor and ANM training** | Karnataka | R | As above | all | Specialist +general hospital (government). | PC | E&T - PC | as above | as above | as above | as above | as above |
| **GOI DMHP- Gulbarga - PC doctor and ANM training (in-depth case study)** | Karnataka | R | As above | all | Specialist +general hospital (government). | PC | E&T - PC | as above | as above | as above | as above | as above |
| **RINPAS - Community mental health programme (CMHP) - PC doctor training (for DMHP)** | Jharkhand | R | 2002-now | all | Specialist hospital (government). | PC | E&T - PC | One-time training only | PC doctor: 15 days training to diagnose, treat +/- refer | Psychiatrists: training, clinical work | Nodal officer (psychiatrist) | Director of the department of health and family welfare |
| **Karuna Trust - PC doctor/ ANM training (for DMHP) (in-depth case study - Gumballi)** | Karnataka | R | 2008-now | all | Specialist/ general hospitals (government) +community (NGO - Karuna Trust) | PC | E&T - PC - Public private partnership | No long term collaboration with specialists but regular contact between doctors and NGO. | PC doctors: trained 1-5 times 3 days to diagnose, treat +/- refer; ANMs (community nurses): trained 1-2 days to identify, refer to PC doctor and basic community support. | Visiting NIMHANS and general hospital psychiatrists: train PC doctors and ANMs +clinical work | NGO mental health coordinator: (usually a general health professional) supervises PC doctors and coordinates training/monitoring of programme | NGO director |
| **GASS-PC doctor training (closed – ie used to exist but no longer in existence)** | Karnataka | R | 2000 only | all | Community (disability NGO-GASS); specialist hospital (individual psychiatrist from NIMHANS) | PC | E&T - PC | One-time training only | PC doctor: diagnose, treat +/- refer (1-3 days training) | visiting NIMHANS psychiatrist: trained local PC doctors - now stopped | GASS coordinator | Head of GASS |
| **SCARF- PC doctor external training (closed)** | Tamil Nadu | R | 1995-2005 | all | CMHS (NGO) | PC | E&T - PC | One-time training only | PC doctor: diagnose, some treat, but most follow-up treatment +/- refer (3 days training) | Psychiatrist: training, clinical work | Psychiatrist | None |
| **CHAD (department of Community Health, Vellore) - PC doctor training (closed)** | Tamil Nadu | R | CHAD founded 1974. Doc training ~1996-2000 | all | General hospital (NGO) | PC | E&T - PC | One-time training only | PC doctor: diagnose, treat +/- refer (3 days training) | Psychiatrist: training, clinical work | Psychiatrist | none |
| **IHBAS (Institute of Human Behaviour and Allied Science) - PC doctor external training (for DMHP) (closed)** | Delhi | R | 2000-2004 | all | General hospital (NGO-academic institution) | PC | E&T - PC | One -time training only | PC doctor: diagnose, treat +/- refer (15 days training)  Private doctors: Saturday 1/2 dayx8 weeks) | Psychiatrist: training, clinical work | Psychiatrist | none |
| **Basic Needs-India (NGO) /Samarthya (NGO) /Samuha (CBO)- certificate training for CBR workers** | Karnataka | R | Samarthya since 1996. MH integration with BNI since 2000s | all | CMHS (Basic Needs NGO) +community (Samarthya - disability NGO) | Community (CBO) | E&T - accredited course | One –time training only | CBR workers: expected to have social worker type responsibilities in the community for disability and mental healthcare | Psychiatrists, PSWs: training, clinical work. | Training coordinator | NGO managers |
| **Saarthak - NGO health worker external training/capacity building** | Delhi | U | 1995-now | all | CMHS (NGO) | Community (other CBO/NGOs) | E&T - accredited course | One -time training only | External LHWs in NGOs: trained in identification and psychosocial interventions relating to the Tsunami and anti-trafficking; also sensitization of development sectors to increase focus on mental health. | Psychiatrists and psychologists: training, clinical work | Psychiatric team | None |
| **VOLCOMH (Volunteers for Community Mental Health)-MH programme: own+ other NGO health worker training+ awareness** | Mizoram | R/U | VOLCOMH founded 1992. MH with Saarthak and Unifem since 2006 | all, substance abuse, HIV | Community (NGO) | Community (other CBO/NGOs) | E&T - accredited courses | One -time training only | External LHWs in NGOs (including Saarthak): trained in identification and psychosocial interventions relating to the HIV, MH, drug abuse. | Psychologist: training, clinical work | Psychologist | None |
| **Chellamuthu Trust - caregiver support groups** | Tamil Nadu | R/U | 2008-now | all | CMHS (NGO) | Self-care +community (support groups) | E&T - caregivers and awareness raising | One -time training only | Caregivers receive 1-3 days training to identify relapse, raise awareness, home coping strategies, networking and forming support group | Psychiatrist: training, clinical work | Psychiatrist | None |
| **Antara - caregiver training** | West Bengal | U | Antara foundd 1982. Caregiver training since 1990s | all | CMHS (NGO) | Self-care +community (caregivers) | E&T- caregivers | One -time training only | Caregivers receive 1 day training to identify relapse, medication adherence and coping strategies. | Social workers: training, clinical work | Social workers | Psychiatrists |
| **Ashadeep - caregiver manual** | Assam | R | Ashaddep founded 1996. Rehab/manual since 2007-8. | all (homeless) | CMHS (NGO) | Self-care +community (caregivers) | E&T -caregivers | One -time training only | Caregivers given a self-help manual to identify relapse, medication adherence and coping strategies. | n/a | No training | n/a |
