# Supplementary material for: Human resources and models of mental healthcare integration into primary and community care in India: Case studies of 72 programmes
Source: PLoS One. 2017 Jun 5;12(6):e0178954. doi: 10.1371/journal.pone.0178954 (PMC5459474; doi:10.1371/journal.pone.0178954)
Supplement: S3 Table — (DOCX) [file pone.0178954.s005.docx]

S3 Table. Characteristics of identification, referral and sensitisation programmes.

| **Programme** | **State** | **Urban/ rural** | **Length of programme** | **MD** | **Specialist/ support platform** | **PHW platform** | **Form of collaboration** | **Level of specialist/ non-spec-ialist colla-boration** | **Roles and training of PHWs/ community** | **Roles of specialists** | **Training coordination and delivery** | **Training/ supervision for coordinator** |
| --- | --- | --- | --- | --- | --- | --- | --- | --- | --- | --- | --- | --- |
| **Richmond Fellowship Society/Siddlaghatta - (closed)** | Karnataka | R | 2004-2009 | all | CMHS (NGO) | PC | Replacement&Referral (R&R) - PC training | One -time training only | PC doctor, ANM, Anganwadi: a few days training to identify and refer cases to RFS outreach clinics | Psychiatrist: training, clinical work | Psychiatrist | None |
| **Manasa Nursing Home - PC doctor external training (closed)** | Karnataka | U | Manasa since 1979. PC doc training in 2000s. now stopped. | all | Specialist hospital (private) | PC | R&R - PC training | One -time training only | PC doctor: Identify and refer (1-3 days training) | Psychiatrist: training, clinical work | Psychiatrist | None |
| **GASS - Health assistants, anganwadi, ANM, self help groups, teacher training** | Karnataka | R | Training done in 2010. ? ongoing. | all | Community (GASS-disability NGO) + CMHS (Basic Needs UK - NGO) | PC +community | R&R - PC training | One -time training. regular interaction with community staff for MH and disability | ANMs: 5 days training (ANMs) and  Anganwadis,SHG members: 2 days training in identification and referral and sensitisation (street plays). | Basic Needs and GASS coordinators/ leaders (PSW/MSW backgrounds respectively) training, management, leadership | Basic Needs and GASS coordinators and leaders | None |
| **Chellamuthu Trust - DMHP PC doctor training + VHNs, Anganwadis** | Tamil Nadu | R | 2008 | all | CMHS (NGO) | PC + community | R&R - PC training | One -time training only | PC doctor (15 days training) and other PC/community staff (3 days training) identify and refer to psychiatrist at outreach camps. | Psychiatric team (psychiatrist, psychologist, PSW) do regular outreach camps. Train PC doctors and other PC staff | Psychiatric team | Head of NGO |
| **TTK Ranganathan - external training (medical+ nursing students)** | Tamil Nadu | U | ? since 2000s | substance abuse | Specialist hospital (NGO) | PC/general care | R&R - PC training | One -time training only | PC doctor: identify, preliminary diagnosis and refer cases of substance abuse | PSW: training, clinical work | PSW | None |
| **SCARF (Schizophrenia Research Foundation) - telemedicine (closed)** | Tamil Nadu | R | SCARF founded 1984. Telemedicine ~2005-2009 | SMDs | CMHS (NGO) | PC | R&R - PC training | One -time training, with no supervision but contact to organise teleconference sessions | PC doctor trained 1-2 days to identify and refer to psychiatrist and organise telemedicine sessions. | Psychiatrists: telemedicine consultations. Train PC doctor. | Psychiatrist | None |
| **Banyan- PC doctor external training (for DMHP)** | Tamil Nadu | R | 2011-now | all | CMHS (NGO) | PC | R&R - PC training | One -time training only | PC doctors trained to identify and refer (1-3 days training) | Banyan psychiatrist helped train PC doctors | Banyan-BALM training coordinator | BALM director |
| **AIIMS (All India Institute of Medical Sciences) - Kashmir Earthquake PC staff training (closed)** | Kashmir/Delhi | R | 2005 only | all | General hospital (academic government) | PC | R&R - PC training | One -time training only | PC doctor: detect, refer and follow-up patients (up to one week training)  PC paramedics (LHWs, pharmacists and nurses): first aid, assist doctors in day-to-day care, dispense medications, counselling.  Training: 1 day (lecture, case vignettes) and case discussion, Supervision: PC doctor. | Psychiatrist: training, diagnosis and treatment | Experiential learning within psychiatrist-led outreach clinic (humanitarian relief dispatch) | None |
| **Basic Needs UK/ NBJK- ANM/Asha training** | Jharkhand | R | 2000-now | all | CMHS (NGO) | PC+ community | R&R - PC training | One -time training but regular contact re disability/ mental health work | ANM/ASHA in programme locality: 1 day training: identify, refer, follow-up, awareness raising | Basic Needs coordinator (psychology/PSW): training, regular contact with ANM/ASHA, management, leadership | Assistant mental health coordinator organises training; Basic needs coordinator delivers it | NBJK and BN-UK directors |
| **Banyan BALM - external NGO training/ capacity building** | Tamil Nadu | R | 2008-now | all | CMHS (NGO) | Community (other CBO/NGOs) | R&R -community training | External organisation one-time training only | HWs from other NGOs: sensitised to MH and to identify and refer (1 day training) | Psychologists, psychiatrists and PSWs: training, clinical work | Training coordinator (BALM) and delivery by specialists | Director of BALM |
| **Bapu Trust - external NGO training** | Maharashtra | U | Bapu Trust since 1999. Training since mid 2000s | all | CMHS (NGO) | Community | R&R -community training | External organisation one-time training only | HWs from other NGOs: 1 day training: identify, refer | Psychologist: training, clinical work  Art therapist: prevention/promotion activities, run support groups | Psychologist, art therapist, family counsellor | NGO director, assistant director |
| **Institute of Psychological Health/ Maitra - NGO and corporate external training** | Maharashtra | U | IPH founded 1990. Maitra since 1998 | all | CMHS (NGO) | Community | R&R -community training | External organisation one-time training only | HWs from other NGOs/corporate sector: trained (1 day) to identify, refer and support patients/families | Psychologist: training, clinical work | Psychologist | NGO director |
| **ANT - external training to NGOs** | Assam | R | 2008-now | all | Community (NGO) | Community | R&R -community training | External organisation one-time training only | HWs from other CBOs and NGOs: trained to include mental health into their development or health initiatives | None | Coordinator (MSW) | NGO head (general physician) |
| **TTK Ranganathan - external training** | Tamil Nadu | R/U | Since 2000s. | substance abuse | Specialist hospital (NGO) | Community | R&R -community training | External organisation one-time training only | Police, clergy, community workers: identify and refer (1 day training) | Psychologist and PSWs: training, clinical work | Specialists | Head of NGO |
| **SNEHA - external training** | Tamil Nadu | U | 1986-now | all, main-ly sui-cide prevention | CMHS (NGO) | Community | R&R -community training | External organisation one-time training only | Police, other community workers: sensitise to MH and to identify and refer (1 day training) | Psychiatrist/ SNEHA director: training, clinical work | Psychiatrist/ SNEHA director and experienced volunteers/clinical coordinators | None |
| **Muktangan Mitra - community external training** | Maharashtra | R/U | Muktangan Mitra founded 1986. Training since 1990s | substance abuse | Specialist hospital (NGO) | Community | R&R -community training | External organisation one-time training only | Police, rehab/de-addiction centres, prison officers, traffic officers: 1 day training on identification and referral for HIV, stress management, alcohol/drug abuse | Psychiatrist, psychologist, PSW: training, clinical work | Specialists | NGO director |
| **Banyan/panchayat academy training** | Tamil Nadu | R | 1994- now | all | CMHS (NGO) | Community (CBO) | R&R -community training | Partnership of organisations, shared decision making about programme direction | Panchayat leaders identify and refer to Banyan. Trained by coordinator and Banyan specialists (1 day) | Banyan leader (PSW): receives referrals, trained Panchayat Academy head and panchayat leaders. Maintains contact with coordinator. | Panchayat Academy head (coordinator): coordinates programme and ensures adequate people referred to Banyan. monitors the programme. | Ad-hoc training, established collaborative rapport between Panchayat Academy head and Banyan |
| **SNR Hospital Kolar/ Murgamalla dargah camps** | Karnataka | R |  | Severe mental disorders (SMDs) | General hospital (government) | Community (religious institution) | R&R -community training | Minimal collaboration | Assistant and religious leaders: ad hoc training to identify and refer to psychiatrist during camps | Psychiatrist: outreach camps fortnightly. He also surveys the dargah to check on adequate care (no chaining) for mentally ill. | Assistant: organise psychiatric camps and bring patients to them; Supervisor: administrative coordination | Informal training, no supervision. |
| **SACRED - advocacy/awareness campaigns** | Andhra Pradesh | R |  | all | Community (SACRED - disability NGO) | Community | R&R - campaigns | Intermittent campaigns | LHWs and local federations and self-help groups (training from BNI and NIMHANS) help deliver campaigns to general population (also do social-worker-like duties). | Psychiatrists train federation members | SACRED training coordinator organises these campaigns and delivers them with PHWs. | SACRED managers |
| **Saarthak - volunteer-led campaigns** | Delhi | U | 1995-now | all | CMHS (NGO) | Community | R&R - campaigns | Intermittent campaigns | 200 community volunteers (1 day training) support specialist team for information/education campaigns to general population and advocacy (as well as for rehabilitation). | Psychiatrist, psychologists: train and network with volunteers | NGO specialists | Psychiatrists |
| **Ashadeep - distribution poster/leaflets** | Assam | R | 2007-now | all (homeless) | CMHS (NGO) | Community | R&R - campaigns | Ongoing campaign | HWs from linked CBOs/NGOs: regularly disseminate pamphlets and posters for general population. | None | Community resource centre coordinator (training centre). | Ashadeep co-director |
| **Manasa Arts - awareness raising+films** | Karnataka | U | 1990s-now | all | Specialist hospital (private) | Community | R&R - campaigns | Intermittent campaigns | General population: exposed to MH awareness. | Psychiatrist: talks, interviews and featurefilms to raise awareness about MH | Specialist-led | None |
| **Richmond Fellowship Society - campaigns/NGO training** | Karnataka | R | 2004-now | all | CMHS (NGO) | Community | R&R - campaigns | Intermittent campaigns | General population: exposed to MH awareness | Psychiatrist: training, awareness raising events, lead campaigns. | Specialist coordinator: organises and conducts some awareness-raising events | None |
| **VOLCOMH - college campaigns** | Mizoram | R/U | VOLCOMH founded 1992. MH with Saarthak and Unifem since 2006 | all, substance abuse, HIV | Community (NGO) | Community | R&R - campaigns | Intermittent campaigns | Colleges students/young professionals: exposed to MH awareness campaign to help with self or other identification and referral. | Clinical psychologist/ leader lead campaigns. | Leader (clinical psychologist) | None |
