# Supplementary material for: Human resources and models of mental healthcare integration into primary and community care in India: Case studies of 72 programmes
Source: PLoS One. 2017 Jun 5;12(6):e0178954. doi: 10.1371/journal.pone.0178954 (PMC5459474; doi:10.1371/journal.pone.0178954)
Supplement: S4 Table — (DOCX) [file pone.0178954.s006.docx]

S4 Table. Characteristics of community outreach programmes.

| **Programme** | **State** | **Location** | **Length of programme** | **MDs** | **Specialist platform** | **PHW platform** | **Level of PHW/community and specialist interaction** | **Stepped/matched or single intervention** | **PHWs: background and roles** | **Specialists: background and roles** | **Care manager/coordinators: background and roles** | **Training + supervision of care manager/ care coor-dinator** | |
| --- | --- | --- | --- | --- | --- | --- | --- | --- | --- | --- | --- | --- | --- |
| Community outreach models in specialist programmes with care coordinator | | | | | | | | | | | | | |
| **Banyan-Urban Mental Health Programme (outreach clinic with PHW support)** | Tamil Nadu | U | 2007-now | all | CMHS (NGO) | CMHS/community (clinic) | Regular organised supervisory meetings between psychiatrist, social workers and volunteers | Matched care determined by PHW-delivered triage | Volunteers (LHWs) and social workers: Joint outreach clinics with psychiatrist. Support specialist by doing triage, history taking and also providing support/advice. Refer to psychiatrist only if necessary. Patient contact at clinic only (not home-based) | Psychiatrist: diagnosis, treatment, supervise volunteers (Outreach clinics ) | Social workers: Coordinate activities (clinical roles under 'PHW roles') | Supervised by psychiatrist | |
| **MHAT (Mental Health Action Trust) (outreach clinic with PHW support)** | Kerala | R | 2008-now | Chronic mental disorders (schizophrenia, bipolar, severe personality disorder) | CMHS (NGO) | community | Regular organised supervisory meetings between psychiatrists, psychologists and volunteers. | Matched care determined by psychiatrist | Care volunteers (LHW): existing palliative care volunteer with added MH respons-ibility; weekly home visits; assigned one patient for life, psychosocial education and family support;  clinic volunteers: screening; home care management volunteers: help psychologist +/-psychiatrist (home visits or nursing home) with palliative and psychiatric needs. | Specialist team (psychiatrist, psychologist, PSW): diagnosis, treatment, follow-up (outreach clinics). Psychologists also do home visits. | Care volunteers: (LHW with certificate). Clinical roles as under 'PHW'. Liaise between community, other LHWs and specialists  coordinator of clinic volunteers. | 6 month mental health training+diploma. Intensive supervision for psychologists and psychiatrist (weekly phone call). | |
| **SCARF-COPSI (Care for people with Schizophrenia in India – RCT) (PHWs as lay counsellors)** | Tamil Nadu | R | 2009-2012 | Schizophrenia | CMHS (NGO) | community | Regular organised supervisory meetings between psychiatrists, care managers and LHWs. | Matched to Stigma intervention by psychiatrist | Community level workers (LHWs): complementary roles to specialists (Home visits): identification/ referral, psychoeducation, stigma interven-tion, general support, raising awareness, referral if necessary. Supervised by coordinators (weekly), doctors (fortnightly), and by supervisors (3 monthly) | Psychiatric team (PSW, psychiatrist, psychologist): diagnosis, treatment, follow-up, (weekly outreach clinics in different loca-tions).Also trial monitor-ing, advocacy + networking | Coordinator (PSW+psychologist back-ground)Supervise PHWs, liaise between community, LHWs+specialists. Coordinate programme, network with agencies. No clinical roles. | Supervised by psychiatrist. | |
| **Bapu Trust-Seher urban MH programme (PHWs as lay counsellors)** | Maharashtra | U | Bapu trust since 1999. Seher since 2004. | all | CMHS (NGO) | Community | Regular contact, meetings and case conferences for support and exchange of patient information | Matched care by specialist team but also some identification/awareness raising done by LHWs | LHW counsellors ie field workers and peer supporters (subtype of fieldworker who provide intensive 24/7 support for those in need): identification, referral services, psychoeducation, inclusion support (social retraining and disability mainstreaming), bring patients to camp, counselling, corner meetings. Generalist doctor: employed just for outreach clinics to rule out organic disorders.  Training of non-formal care givers | Specialist team (psychologists, PSW, art-therapists, homeopath): outreach clinics including psychotherapies. Psychiatrist does field worker training. | LHW counsellor called 'fieldworker' - recovered patients: Liaise between patients and specialist. Care roles as under 'phw roles' | Supervised by psychologists and leader/coordinator. Also trained by psychiatrist, psychologist PSWs and coordinators | |
| **Muktang Mitra-outreach rural (PHWs as lay counsellors)** | Maharashtra | R | MM founded 1986. Outreach programme more recent (?1990s) | substance abuse | Specialist hospital (NGO) | Community | Regular organised supervisory meetings between specialists, coordinators and volunteers | Matched care determined by psychiatrist | Volunteers (LHWs): (1 month training or more; some have CBT/REBT training) support specialist care by providing counselling, psychosocial support (outreach clinics); also receive calls in call centre (see below); Caregivers: trained by volunteers. | Psychiatrist and psychologist: diagnosis, treatment, supervise volunteers (Outreach clinics). | Coordinator (clinic-based mental health paraprofessional). Coordinates and supervises volunteers and counsellors. Also has treatment/care roles within NGO clinical services. | Training and supervision by specialists (of all PHWs). | |
| **Saarthak PACT (reintegration project for people recoverin from severe mental illness) (PHWs as lay counsellors)** | Delhi | R | 1995-now | Severe or enduring mental disorders | CMHS (NGO) | Community | Regular organised supervisory meetings between psychiatrists, psychologists and facilitators. | Stepped care - psychosocial support (prim-ary fa-cilitator; coun-selling(secondary fa-cilitator,referral to psychiatrist | Primary facilitator (graduates or recovered users)): psychosocial support, befriending, activities; secondary facilitator (with 1 year Saarthak diploma): group leaders, counselling and supervise/peer support with primary facilitators. (Home visits) | Psychiatric team (PSW, psychiatrist, psychologist): diagnosis, treatment including therapies. (outreach clinics) | Secondary facilitators: (diploma graduates). lead team, liaise between primary facilitators, community and specialists.  Psychologists coordinate the programme | 1 year diploma course. Supervision by therapists and psychiatrists. | |
| **VOLCOMH-outreach programme (PHWs as lay counsellors)** | Mizoram | R | VOLCOMH founded 1992. MH with Saarthak and Unifem since 2006 | all, substance abuse, HIV | CMHS (NGO) | Community | Regular organised supervisory meetings between psychologists and outreach workers. | Stepped care - first home-based support (LHWs), then psychologist, then refer to psychiatrist. | Peer educators (PE's)(recovered users- LHWs): identification, referral, follow-up, some counselling, psychosocial support, awareness raising (3 days training in house). Outreach workers (LHWs): supervise PE's and do livelihood/ benefits work with clients. All PHWs involved in HIV and substance use care too. (Home visits). | Clinical psychologists trained by programme coordinator/leader and perform community-based clinics at VOLCOMH centre. No psychiatrists (can refer to government psychiatrist). | Outreach workers: (graduates/SWs or experienced users/previous peer educators (PE's). clinical roles as under 'PHWs'. Liaise between PE's and psychologists/ head of VOLCOMH. Supervise PE's including joint visits every week (as do psychologists). | Trained for 5 days. Significant initial in-house and ongoing training for ORWs and PE's. Supervised by psychologists | |
| **SNEHA- helpline** | Tamil Nadu | R/U | 1986-now | all | CMHS (NGO) | CMHS | Regular meetings between psychiatrist and coordinators and coordinators with volunteers. | Single PHW-led intervention (befriending) | Volunteers (LHWs). 40 days training + ongoing training. provide emotional first aid, and keep records of discussions. (Call centre) Also do fundraising. | Psychiatrist: supervision, training, external training, overall programme coordination. | Experienced volunteers (non-health lay background (LHW)). Train volunteers, Coordinate and supervise volunteer call receivers. Also clinical roles as under PHW roles. | Both by psychiatrist leader. | |
| **Maitra-helpline** | Maharashtra | R/U | 1998-now | all | CMHS (NGO) | CMHS | Regular meetings between coordinator, care managers and volunteers | Single PHW-led intervention (befriending) | Volunteers (LHWs). 3-5 days training and ongoing training every 2-3 months. provide emotional first aid. (Call centre). | Psychologist and PSW. supervise and train volunteers. | Psychologist and PSWs: coordinate, supervise and train volunteers and some also involved in receiving calls. (also have other clinical roles with the NGO (Maitra). | Supervised by programme coordinator/ counsellor (non-health background initially). | |
| **Muktang Mitra-helpline** | Maharashtra | R/U | 1986 | substance abuse | Specialist hospital (NGO) | CMHS | Moderate communication between specialists and coordinators, best contact between coordinators and volunteers | Single PHW-led intervention (support and crisis intervention) | Volunteers (LHWs): 1 month training, some have additional REBT or CBT training. provide support, advice and minimal counselling. (Call centre) | Psychiatrist, psychologist: supervise and train coordinators | Coordinator (mental health paraprofessional): supervises/monitors volunteers calls. Trains volunteers. Also has treatment/care roles within NGO clinical services | Psychiatrist and coordinator | |
| **Uduvam Ulangal- rescue operation (for shelter)** | Tamil Nadu | R | ?2000s | all | General hospital (individual psychiatrist) | community | Specialist team communication good | Matched care determined by psychiatrist | LHWs (lay counsellor/social worker): part of outreach team. Supportive role to the specialist for psychosocial support | Psychiatrist: visit hot spots in community with LHWs. Also diagnosis and treatment | Coordinator (graduate/PHW): main role administrative coordinating CHAD and government hospital activities. Minor roles in PHW support. Also does patient family reintegration | Psychiatrist | |
| **Mission Ashra-rescue operation (for care unit)** | Orissa | R | 2000-now | all (homeless) | Specialist hospital (NGO) | PHWs from specialist hospital (nurses and general social workers) | Minimal interaction between psychiatrist and PHWs | Matched care determined by psychiatrist | Pharmacist, nurse, social worker: part of outreach team. Supportive role but also do counselling and help with children, refer to the care unit/rehab and do resilience training for community/families | Psychiatrist, psychologist: team (+PHW social worker) visit hot spots in community. Psychiatrist: diagnosis and treatment | Psychiatrist: very little training and support for PHWs | None | |
| Vocational rehabilitation (not a first level access but community based service) | | | | | | | | | | | | |  |
| **Samuha (CBO)/ Samarthya (NGO)/ Basic Needs India (CMHS) -vocational rehabilitation** | Karnataka | R | Samarthya since 1996. MH integration with BNI since 2000s | SMDs | CMHS (NGO) + community (disability NGO) | Community (CBO)+ self-care | Minimal contact with specialists (only available for referral). Regular organised contact between coordinators and PHWs | Matched care determined by psychiatrist (horticulture or tailor training) | Recovered patients: horticultural, tailoring trainers (community centre) | No specialists involved in this service apart from referring to local psychiatrists | Several Samuha and Samarthya coordinators including a horticultural coordinator (experienced CBR worker) who supervise and train PHWs | Supervision and training by Basic Needs coordinators and Samarthya managers | |
| **Banyan - day care centre (rehabilitation)** | Tamil Nadu | U | 2008- now | SMDs | CMHS (NGO) | Self-care | Regular weekly interaction between psychiatristand coordinator | single intervention (client-led decision (computer or crafts training) | Vocational trainers in IT and arts/crafts: (Recovered patients (health care worker or skilled instructors): Also follow-up patients with regards to their medication effect (in a centre) | Psychiatrist: weekly outreach clinics. | Coordinator (non-health graduate background) coordinates activities and supervises vocational trainers | Hierarchy of training and supervision: psychia-trist, coor-dinator, vocational trainers | |
| **Chellamuthu Trust – 5 vocational rehabilitation units** | Tamil Nadu | U | 1996-now | SMDs | CMHS (NGO) | Residential placement (NGO) aiming for Self-care | Contact of psychiatrist with PHWs at outreach clinics | Matched care determined by psychiatrist (vocational training) | LHWs/recovered patients (vocational instructors): different according to unit eg microenterprise, agro-business, tailoring and arts/crafts. Part of holistic psychosocial rehabilitation including specialist support | MDTs including psychiatrist/ outreach clinics – medication/clinical review 3 monthly; creative therapist, occupational therapist. | Psychiatrist: oordination and supervision. | None | |
| **Saarthak - vocational rehabilitation (closed)** | Delhi | U | 1995-2000? | SMDs | CMHS (NGO) | Residential placement (NGO) aiming for Self-care | Contact of psychiatrist with PHWs at outreach clinics | Matched care determined by psychiatrist (vocational training) | Recovered patients: tailoring and arts/crafts trainers (in a centre) | Psychiatrist: outreach clinics | Psychiatrist: oordination and supervision. | None | |
| **Banyan-reintegration programme at Adaikalam (rehabilitation)** | Tamil Nadu | R/U | 1994-now | SMDs | CMHS (NGO specialist unit) | Community + self-care | Visiting team have substantial contact and patient information sharing | Matched care determined by psychiatrist (reintegration into families) | Reintegration volunteers (non-health workers) in community: reintegration of family member. Family support (home visit). Supervised and trained by Adaikalam outreach team | Psychiatrists, clinical psychologists and psychiatric social workers Adaikalam (institution) outreach team: help with the reintegration team activities and support before/ after | Psychiatrist: oordination and supervision. | None | |
